# Supplementary material for: Efficient transgenesis and homology-directed gene targeting in monolayers of primary human small intestinal and colonic epithelial stem cells
Source: Stem Cell Reports. 2022 May 5;17(6):1493–506. doi: 10.1016/j.stemcr.2022.04.005 (PMC9213823; doi:10.1016/j.stemcr.2022.04.005)
Supplement: Document S1. Figures S1 and S2 and Tables S1–S5 [file mmc1.pdf]

Stem Cell Reports, Volume *17*

Supplemental Information

Efficient transgenesis and homology-directed gene targeting in monolayers of primary human small intestinal and colonic epithelial stem cells

Keith A. Breau, Meryem T. Ok, Ismael Gomez-Martinez, Joseph Burclaff, Nathan P. Kohn, and Scott T. Magness

Supplemental Table 1. Electrical pulse parameters for optimization

Sample #	Parameters			Transfection Efficiency			Cell Viability		
	Voltage	Length (ms)	# Pulses	Duo	Jej	Ile	Duo	Jej	Ile
1	0	0	0	Neg	Neg	Neg	High	High	High
2	1400	20	1	SubLow	Low	Moderate	High	High	High
3	1500	20	1	Low	Low	Moderate	Low	High	High
4	1600	20	1	Moderate	Moderate	High	High	High	High
5	1700	20	1	Moderate	High	High	High	High	High
6	1100	30	1	Neg	Neg	Neg	High	High	High
7	1200	30	1	SubLow	SubLow	Low	High	High	Moderate
8	1300	30	1	Low	SubLow	Moderate	High	High	Low
9	1400	30	1	Moderate	Moderate	Moderate	Low	High	Low
10	1000	40	1	Neg	Neg	SubLow	High	High	Moderate
11	1100	40	1	SubLow	Neg	SubLow	Moderate	High	Low
12	1200	40	1	Low	SubLow	Low	Moderate	High	SubLow
13	1100	20	2	SubLow	SubLow	SubLow	High	High	Low
14	1200	20	2	SubLow	SubLow	SubLow	SubLow	High	Low
15	1300	20	2	Moderate	Low	Moderate	SubLow	High	SubLow
16	1400	20	2	High	Moderate	Moderate	SubLow	Moderate	Neg
17	850	30	2	Neg	Neg	Neg	Low	High	SubLow
18	950	30	2	Neg	Neg	Neg	Low	High	SubLow
19	1050	30	2	SubLow	SubLow	Neg	Low	High	Neg
20	1150	30	2	Low	Moderate	SubLow	Low	Moderate	Neg
21	1300	10	3	Neg	Neg	Neg	Moderate	High	SubLow
22	1400	10	3	SubLow	SubLow	Neg	Low	High	SubLow
23	1500	10	3	SubLow	Low	SubLow	Low	High	Low
24	1600	10	3	High	Moderate	Low	SubLow	Moderate	Neg

Supplemental Table 2. Organ donor demographics

Identifier	Patient Sex	Patient Age	Race/Ethnicity	Cause of Death
Donor 1	M	12 years	White	Cerebrovascular/Stroke
Donor 2	M	34 years	Hispanic	Anoxia, Asphyxiation (unknown downtime)
Donor 3	F	62 years	White	Cardiovascular, Death from Natural Causes

Supplemental Table 3. CCTop off-target loci for *OLFM4* gRNA

gRNA Sequence:	ACTTACTTAGATATCTGCAG							
Coordinates	strand	MM	target_seq	PAM	distance		gene name	gene id
chr13:53050828-53050850	+	0	ACTTACTT [AGATATCTGCAG]	GGG	0	E	OLFM4	ENSG00000102837
chr4:95984460-95984482	-	4	TAACACTT [AGATATCTGCAG]	AAG	NA	-	NA	NA
chr6:119536897-119536919	-	4	AGTAGGTT [AGATATCTGCAG]	TGG	NA	-	NA	NA
chr10:1497775-1497797	+	4	CATTCCTG [AGATATCTGCAG]	AAG	28840	I	ADARB2-AS1	ENSG00000205696
chr4:158333757-158333779	+	4	ATCTATGT [AGATATCTGCAG]	GGG	18287	I	RXFP1	ENSG00000171509
chr12:105189620-105189642	-	4	GTTTAGTA [AGATATCTGCAG]	TGG	130	I	APPL2	ENSG00000136044
chr2:127745290-127745312	+	4	GGTTAATC [AGATATCTGCAG]	CAG	7236	I	WDR33	ENSG00000136709
chr5:61465479-61465501	-	3	ACTAATTT [GGATATCTGCAG]	TGG	7180	I	ZSWIM6	ENSG00000130449
chr2:219136091-219136113	-	4	ACAAAATC [AGATATCTGCAG]	GGG	10431	I	NHEJ1	ENSG00000187736
chr3:115685944-115685966	+	4	ATTTTATC [AGATATCTGCAG]	CGG	9334	I	GAP43	ENSG00000172020
chr21:21940614-21940636	-	4	TTTTACCT [TGATATCTGCAG]	TAG	7015	I	AP000472.3	ENSG00000227075
chr10:114724009-114724031	+	4	ATTCATTT [TGATATCTGCAG]	GAG	33375	-	PPIAP19	ENSG00000228169
chr18:47316063-47316085	+	3	ACTTCATT [AAATATCTGCAG]	TGG	30266	I	CTD-2130O13.1	ENSG00000267761
chr6:81765343-81765365	-	4	ATTTGCCC [AGATATCTGCAG]	CAG	840	-	RP5-991C6.2	ENSG00000219702
chr3:128045637-128045659	+	4	ACAGATTT [TGATATCTGCAG]	AGG	5982	-	SEC61A1	ENSG00000058262
chr14:42710344-42710366	-	4	ACAGATTT [CGATATCTGCAG]	GGG	6689	-	CTD-2307P3.1	ENSG00000258394
chr4:144546999-144547021	-	4	ACAGATTT [TGATATCTGCAG]	AGG	25217	-	KRT18P51	ENSG00000250504
chr6:101110566-101110588	+	4	ATTTAGAA [AGATATCTGCAG]	GAG	NA	-	NA	NA
chr13:39413026-39413048	-	4	ATCTACTG [TGATATCTGCAG]	AAG	34500	I	LHFP	ENSG00000183722
chr2:56417840-56417862	-	4	AGAGACTT [AGTTATCTGCAG]	TAG	31667	-	CCDC85A	ENSG00000055813

Supplemental Table 4. Primers used in this study

Primer Name	Oligo Sequence (5' to 3')	Purpose
KB085_Olfm4-ScF2	CCGTACTATGAACACCAGAACAG	PCR amplification of *OLFM4* gRNA target region for sequencing
KB086_Olfm4-ScR2	CGCCATACTGAGTTTTAAGTTCC	PCR amplification of *OLFM4* gRNA target region for sequencing
KB026_Olfm4-SqF1	AGCATTAACTATAACCCTTTTGAC	Sequencing primer for *OLFM4* gRNA target region
KB069_Olfm4-5HR-F2	tggccgattcattaatgcagGACTGAATGCACTGAACTAAGAGG	Amplification of OLFM4 5' homology region
KB038_Olfm4-5HR-R	tcgaatagcttggctgcaggAGATATCTAAGTAAGTGGAGAAGAC	Amplification of OLFM4 5' homology region
KB039_Olfm4-3HR-F	ttaggtccctcgaggggatcGCAGGGGTGTCTAAAAGTGTG	Amplification of OLFM4 3' homology region
KB040_Olfm4-3HR-R	cctcttcgctattacgccagAAGGTTTCCAACTACTGCACTG	Amplification of OLFM4 3' homology region
KB045_emGFP-F	TCGACCTGCAGCCAAGCTATTCGAATTCCCCCCCCCCCTAACGTTACTG	Amplification of emGFP coding sequence
KB046_emGFP-R	TGATCGGAATTGGGCTGCAGGAATTCTTACTTGTACAGCTCGTCCATGC	Amplification of emGFP coding sequence
KB081_IRES-SqR1	TAACATATAGACAAACGCACACC	Screening for OLFM4-emGFP desired integration event
KB084_bGHpA-SqF1	ATGCGGTGGGCTCTATGG	Screening for OLFM4-emGFP desired integration event
KB097_Olfm4-5LR-F1	CTGTAGAGCTGACATTGGGTTTGC	Screening for OLFM4-emGFP desired integration event
KB098_Olfm4-3LR-R1	AGGTAGCCATTCTCCCAACCTC	Screening for OLFM4-emGFP desired integration event

Supplemental Table 5. Medias and buffers used in this study

L-WRN Collection Medium

Reagent	Concentration	Vendor / Cat#
Adv. DMEM/F12	78% v/v	Gibco 12634010
Fetal Bovine Serum	20% v/v	VWR Premium 97068-085
GlutaMax	2 mM (1X)	Gibco 35050061
Pen/Strep	1X	Gibco 15070063

2x hISC Basal Medium

Reagent	Concentration	Vendor / Cat#
Adv. DMEM/F12	89% v/v	Gibco 12634010
B-27 Supplement	4% v/v	Gibco 12587001
Nicotinamide	10 mM	Sigma-Aldrich N0636
HEPES	20 mM	Corning 25-060-Cl
Glutamax	2 mM (1X)	Gibco 35050061
Pen/Strep	1X	Gibco 15070063
N-Acetylcysteine	1.25 mM	Sigma-Aldrich A9165
Primocin	50 ug/mL	Invivogen ant-pm-05
SB202190	3 uM	Peprotech 1523072
mEGF	50 ng/mL	Peprotech 315-09
Gastrin	2 nM	Sigma-Aldrich G9145
Prostaglandin E2	10 nM	Peprotech 3632464

Isolation Buffer

Reagent	Concentration	Vendor / Cat#
Na_2HPO_4	5.6 mM	Sigma S7907
KH_2PO_4	8.0 mM	Sigma P5655
NaCl	96.2 mM	Sigma S5886
KCl	1.6 mM	Sigma P5405
Sucrose	43.4 mM	Fisher BP 220-1
d-sorbitol	54.9 mM	Fisher BP439-500
Y27632	100 uM	Selleck Chemical S6390

Differentiation Medium

Reagent	Concentration	Vendor / Cat#
Adv. DMEM/F12	89% v/v	Gibco 12634010
HEPES	10 mM	Corning 25-060-Cl
Glutamax	2 mM (1X)	Gibco 35050061
Pen/Strep	1X	Gibco 15070063
N-Acetylcysteine	1.25 mM	Sigma-Aldrich A9165
Primocin	50 ug/mL	Invivogen ant-pm-05
mEGF	50 ng/mL	Peprotech 315-09
A83-01	500 uM	Sigma-Aldrich SML0788

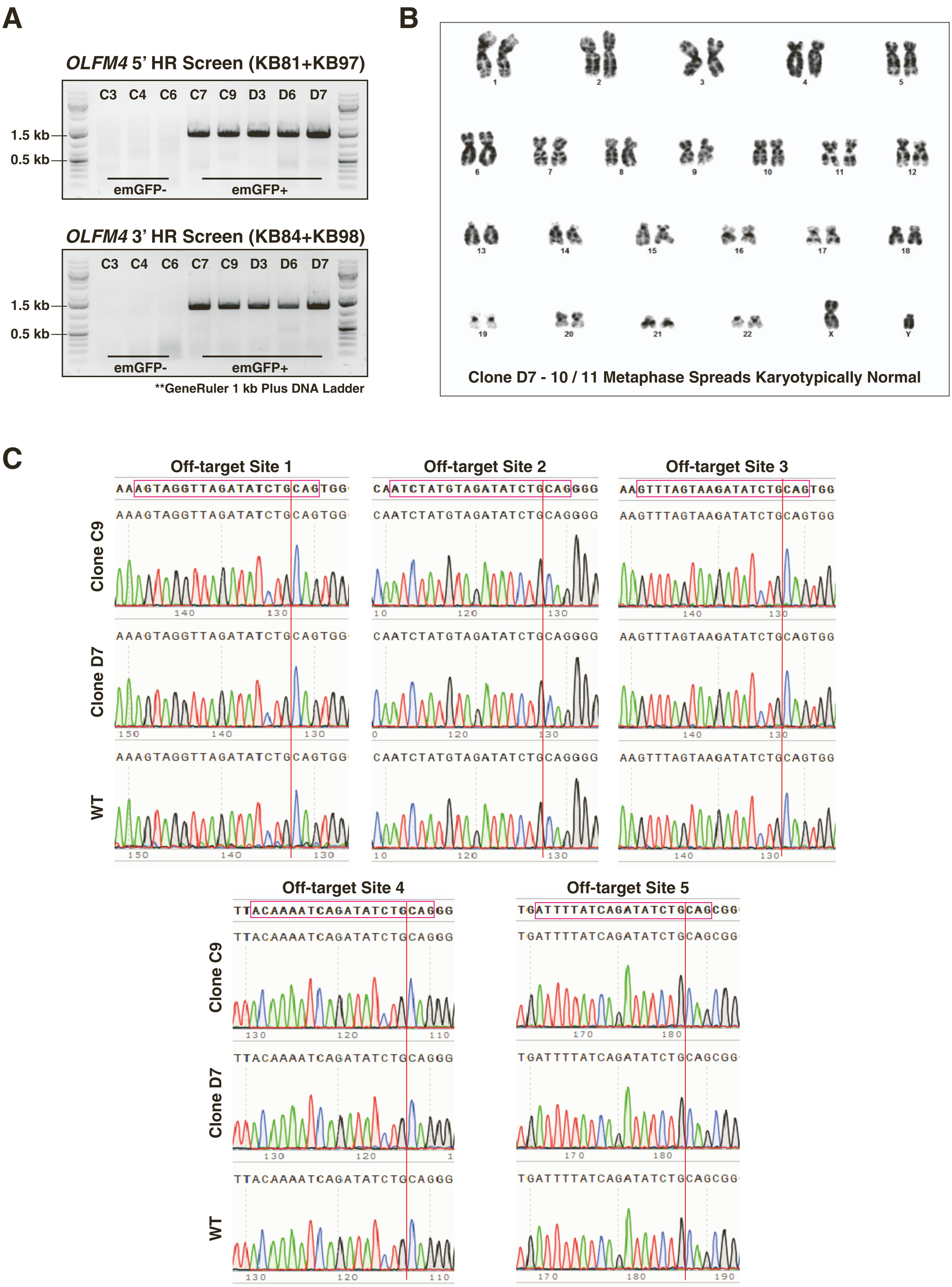

Supplemental Figure 1

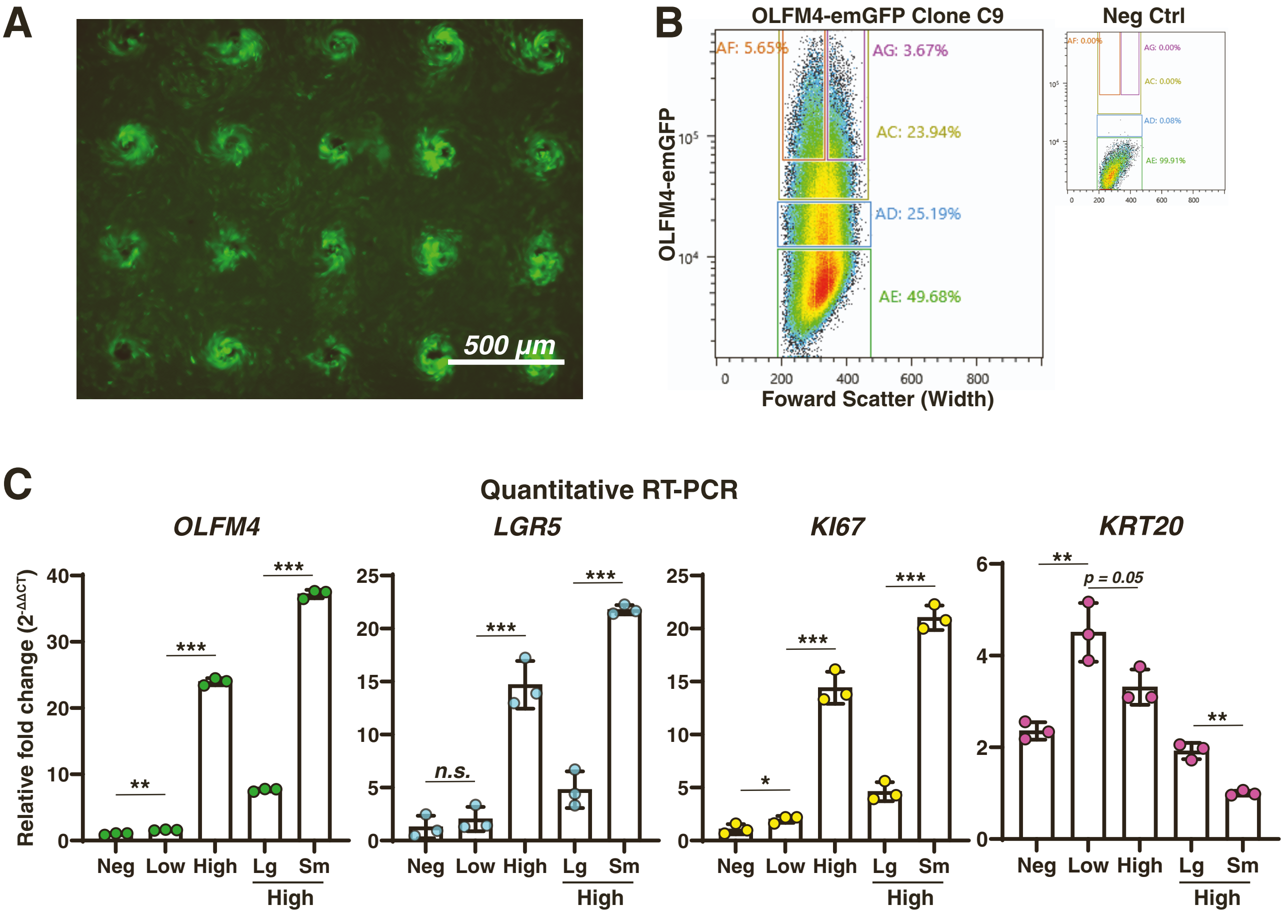

Supplemental Figure 2

Supplemental Table 1, related to Figure 1. Electrical parameters used to optimize electroporation efficiency. Twenty-four parameter sets were tested, varying electrical voltage, pulse duration, and pulse number. Transfection efficiency and cell viability of three biological replicates were estimated and binned into 5 categories (Neg, SubLow, Low, Moderate, and High). Condition #5 (1700 V, 20 ms, 1 pulse), highlighted, was used for further experiments.

Supplemental Table 2, related to Figure 2. Demographic details of organ donors used to test biological reproducibility of transfection efficiency.

Supplemental Table 3, related to Figure 5. CCTop output of the selected *OLFM4*-targetting gRNA, showing DNA sequences throughout the genome with homology to the gRNA sequence. First entry is the desired cleavage site in the terminal exon of *OLFM4*. Latter entries are potential off-target cleavage sites, ranked by decreasing predicted risk of cleavage, with mismatched nucleotides shown in red. gRNA was chosen such that all potential off-target sites have at least four mismatches, or three mismatches with at least one mismatch within the 12 bp “seed” region (shown in brackets), proximal to the Cas9 cleavage site.

Supplemental Table 4, related to Figure 5. Primers used for generating TIDE results (top), generating the *OLFM4* targeting plasmid (middle), and validating integration of the IRES-emGFP sequence (bottom).

Supplemental Table 5. Isolation buffer used for crypt isolation, and medias used for subsequent hISC expansion and differentiation.

Supplemental Figure 1, related to Figure 5. (A) PCR validation of IRES-emGFP integration at the *OLFM4* locus. Three emGFP-negative clones were included for a negative control, along with 5 emGFP+ clones. Primers were designed to span the homology arms used for HDR, with one primer in each screen binding to the native *OLFM4* locus outside of the homology regions and the other binding to the IRES-emGFP insert. **(B)** Example metaphase spread for OLFM4-emGFP

clone D7, which is used in the primary figures of the manuscript, demonstrating these cells are karyotypically normal. **(C)** Sequencing results of high-risk off-target cleavage sites of the OLFM4 gRNA. Sites represent all genomic loci with a perfect match of the gRNA seed region (12 bp at 3' end) and a PAM sequence of NGG. Sequencing demonstrates no mutations in either clone used in this study.

Supplemental Figure 2, related to Figure 6. Analysis of a second OLFM4-emGFP clone (clone C9) demonstrates similar results to clone D7, showing **(A)** compartmentalization of emGFP+ and emGFP- zones on a PCM device, **(B)** flow cytometry of PCM-isolated cells, with a gradient of OLFM4-emGFP signal and a separate, smaller OLFM4-emGFP^{high} population, and **(C)** qPCR results of FACS-isolated cell populations demonstrating High^{sm} cells highly express stem cell markers *OLFM4*, *LGR5*, and *KI67*, and poorly express differentiation marker *KRT20*.
