# Supplementary material for: Efficient transgenesis and homology-directed gene targeting in monolayers of primary human small intestinal and colonic epithelial stem cells
Source: Stem Cell Reports. 2022 May 5;17(6):1493–506. doi: 10.1016/j.stemcr.2022.04.005 (PMC9213823; doi:10.1016/j.stemcr.2022.04.005)
Supplement: Document S2. Article plus supplemental information [file mmc2.pdf]

Stem Cell Reports
Resource

OPEN ACCESS

Efficient transgenesis and homology-directed gene targeting in monolayers of primary human small intestinal and colonic epithelial stem cells

Keith A. Breau,[2] Meryem T. Ok,[1] Ismael Gomez-Martinez,[2] Joseph Burclaff,[3] Nathan P. Kohn,[3] and Scott T. Magness[1,2,3,4,*]

[1]Joint Department of Biomedical Engineering, University of North Carolina at Chapel Hill and North Carolina State University, Chapel Hill, NC 27599, USA
[2]Department of Cell Biology & Physiology, University of North Carolina at Chapel Hill, Chapel Hill, NC 27599, USA
[3]Center for Gastrointestinal Biology and Disease, University of North Carolina at Chapel Hill and North Carolina State University, Chapel Hill, NC 27599, USA
[4]Department of Medicine, University of North Carolina at Chapel Hill, Chapel Hill, NC 27599, USA
*Correspondence: magness@med.unc.edu
https://doi.org/10.1016/j.stemcr.2022.04.005

SUMMARY

Two-dimensional (2D) cultures of intestinal and colonic epithelium can be generated using human intestinal stem cells (hISCs) derived from primary tissue sources. These 2D cultures are emerging as attractive and versatile alternatives to three-dimensional organoid cultures; however, transgenesis and gene-editing approaches have not been developed for hISCs grown as 2D monolayers. Using 2D cultured hISCs we show that electroporation achieves up to 80% transfection in hISCs from six anatomical regions with around 64% survival and produces 0.15% transgenesis by PiggyBac transposase and 35% gene edited indels by electroporation of Cas9-ribonucleoprotein complexes at the *OLFM4* locus. We create OLFM4-emGFP knock-in hISCs, validate the reporter on engineered 2D crypt devices, and develop complete workflows for high-throughput cloning and expansion of transgenic lines in 3–4 weeks. New findings demonstrate small hISCs expressing the highest *OLFM4* levels exhibit the most organoid forming potential and show utility of the 2D crypt device to evaluate hISC function.

INTRODUCTION

In humans, the lower digestive track is divided into two organs, the small intestine (SI) and colon, which together orchestrate digestion and uptake of food, oral drugs, water, and electrolytes (Kiela and Ghishan, 2016). The epithelium is a one-cell layer, lines the whole inner surface of both organs, carries out region-specific physiological processes, and creates a strong and selective barrier to luminal contents. The epithelial lining is constantly renewed by actively dividing intestinal stem cells (ISCs) (Barker, 2014; Cheng and Leblond, 1974). ISCs give rise to highly proliferative transit amplifying progenitors that terminally differentiate into absorptive and secretory cell types (Cheng and Leblond, 1974). The mechanisms underlying ISC-driven epithelial renewal in physiology, disease, and injury is an active area of research, and developing improved *in vitro* methods to understand these mechanisms has strong potential to address health conditions affecting the gut.

Until recently, long-term *in vitro* culture of normal human ISCs (hISCs) was only possible via three-dimensional (3D) organoids(Sato et al., 2011). Organoids are spherical epithelial tissue structures typically smaller than 1 mm that are cultured in 3D hydrogels with media mimicking ISC niche signaling (Sato et al., 2009). ISCs in organoids differentiate stochastically or by induction with native factors or small molecules (Sato et al., 2009), providing a highly accurate model of the gut epithelium. However, 3D organoid utility can be limited by complex 3D culture methods, lack of simultaneous apical and basolateral access, and challenging imaging and quantification through the Z-planes of 3D hydrogels.

Recent advances in culturing hISCs as two-dimensional (2D) monolayers offer long-term culture and expansion of hISCs and resolve many of the challenges of 3D organoid cultures (Wang et al., 2017a). The 2D hISC monolayer cultures rely on the same cellular starting material and general media formulations used to generate 3D organoids, but can be grown in larger culture dishes using inexpensive collagen hydrogels, applied to Transwells for simultaneous apical and basolateral access, and easily imaged as cells are cultured on a planar surface. The 2D hISC monolayers can be easily converted to and from 3D organoid cultures (Wang et al., 2017a), differentiated on Transwells to evaluate barrier function (Wang et al., 2019), applied to microfabricated devices that recreate 3D microanatomical features of crypts and villi (Wang et al., 2017b), or applied to 2D crypt microphysiological systems that compartmentalized arrayed ISC and differentiated zones (Kim et al., 2018).

Efficient transfection, transgenesis, and clonal isolation methods using 2D hISC cultures would enhance hISC utility for mechanistic studies, disease modeling, and development of high-content platforms for drug screening, however, methods have only been reported for 3D organoids (Fujii et al., 2015; Kashfi et al., 2020; Matano et al., 2015; Schwank and Clevers, 2016). Compared with other

This is an open access article under the CC BY license (http://creativecommons.org/licenses/by/4.0/).

methods electroporation resulted in the highest transfection efficiencies in colonoids (approximately 30%) (Fujii et al., 2015), and produced up to 75% efficiencies in other hard to transfect cells cultured as monolayers (Jordan et al., 2008), thus, we hypothesized that electroporation could be used to efficiently transfect hISCs cultured as 2D monolayers.

Here we optimize electroporation parameters, apply these parameters to transfect 2D hISC monolayers isolated from six different regions of the human SI and colon, assess transgenesis efficiency for random integration and site-specific CRISPR/Cas9 gene editing, and develop clonal isolation and expansion methods for transgenic hISCs that offer substantially improved throughput over existing methods. We then apply this new method to create an emerald green fluorescent protein (emGFP) reporter gene line to identify hISCs *in vitro* and use these cells to demonstrate the utility of a recently developed 2D microphysiological model of hISC maintenance and differentiation.

RESULTS

A single, high-voltage pulse efficiently transfects hISCs with high viability

The high transfection efficiency of primary cells offered by electroporation often comes at the expense of cell viability (Jordan et al., 2008); thus, we tested electrical field and pulse conditions to identify conditions that supported the highest transfection efficiency and viability in hISCs. hISCs from duodenal, jejunal, and ileal crypts isolated from a single organ donor were expanded as monolayers on collagen-coated six-well culture plates in media containing WNT3A, NOGGIN, and R-SPONDIN3 to support hISC expansion and suppress differentiation (Hinman et al., 2021; Wang et al., 2017a). Low passage ($p < 12$) hISCs were dissociated to single cells, and the Neon electroporation system was used to transfect hISCs with a constitutively expressing enhanced green fluorescent protein (EGFP) plasmid to quantify electroporation efficiency. Twenty-four electroporation conditions (varying voltage, pulse duration, and pulse number) were tested on hISCs from three SI regions (Figure 1A and Table S1).

A semi-quantitative approach was used to rapidly assess and filter transfection efficiencies from the 72 independent electroporations. By visual inspection, hISCs were binned into five categories based on estimated percent of EGFP-expressing cells (i.e., transfection efficiency): negative (no visible GFP), sub-low (<10%), low (10%–30%), moderate (30%–50%), and high (>50%) (Figures 1B–1D). Cell survival was similarly scored by visually comparing cell coverage to an untransfected negative control 48 h after transfection (Figures 1C–1E). One electroporation condition (1700 V, 20 ms, 1 pulse) consistently yielded high transfection efficiency and viability in cells from all three small intestinal regions (Figures 1D and 1E), and this condition was used to quantify transfection efficiency across all six SI and colon regions.

Flow cytometric analysis of transfected hISCs demonstrated robust transfection regardless of hISC anatomical origin, with transfection efficiencies up to approximately 80% (Figure 2A). Dissociated monolayers yielded an average of 1.25 × 10^6 and 0.64 × 10^6 live single cells per 9.6 cm^2 well for SI and colon, respectively (Figure 2B). Thirty-seven independent electroporations over different passage numbers and three donors (Table S2) resulted in a mean transfection efficiency of 43.8% ± 18.8% with 64.7% ± 12.6% survival and no significant differences between SI and colon hISCs (Figures 2C and 2D). A negative correlation was seen between transfection efficiency and hISC passage number ($p = 0.05$; $R^2 = 0.38$), consistent with reports that transfection efficiencies can decline over time in other primary cell lines (de Carvalho et al., 2018; McCoy et al., 2010) (Figure 2E).

New high-throughput methods for transgenic colony isolation from 2D collagen hydrogels

Clonal colony isolation and subsequent expansion is critical for developing transgenic hISC lines. The 2D collagen hydrogels are essential for long-term hISC self-renewal in 2D culture; thus, new methods were developed to achieve these goals (Wang et al., 2017a). To optimize workflows, antibiotic resistant hISC colonies were generated on 2D collagen hydrogels by electroporating a PiggyBAC transposase plasmid with plasmid containing a puromycin resistance gene flanked by inverted terminal repeats. After 3 days of recovery, hISCs were selected with puromycin for 6 days, at which point antibiotic-resistant hISC colonies became visible (Figures 3A and 3B).

To isolate individual clones, a 20-min incubation with collagenase IV proved highly effective at releasing intact hISC colonies (Figure 3C). Liberated colonies were transferred to a 10-cm dish to distribute across a larger surface for ease of clonal isolation. Individual colonies were collected by pipet under brightfield microscopy and transferred to collagen-coated 96-well plates (Figure 3D). Using this approach, 96 colonies could be isolated in approximately 45 min. Four days after isolation, 50.6% ± 19.1% of the isolated colonies attached and expanded as 2D monolayers (Figures 3E and 3F).

PiggyBac transfection methods efficiently generate stable single- and multiple-transgene integration events

Stable transgene integration is commonly used to generate reporter gene and gain-of-function cell lines. To evaluate

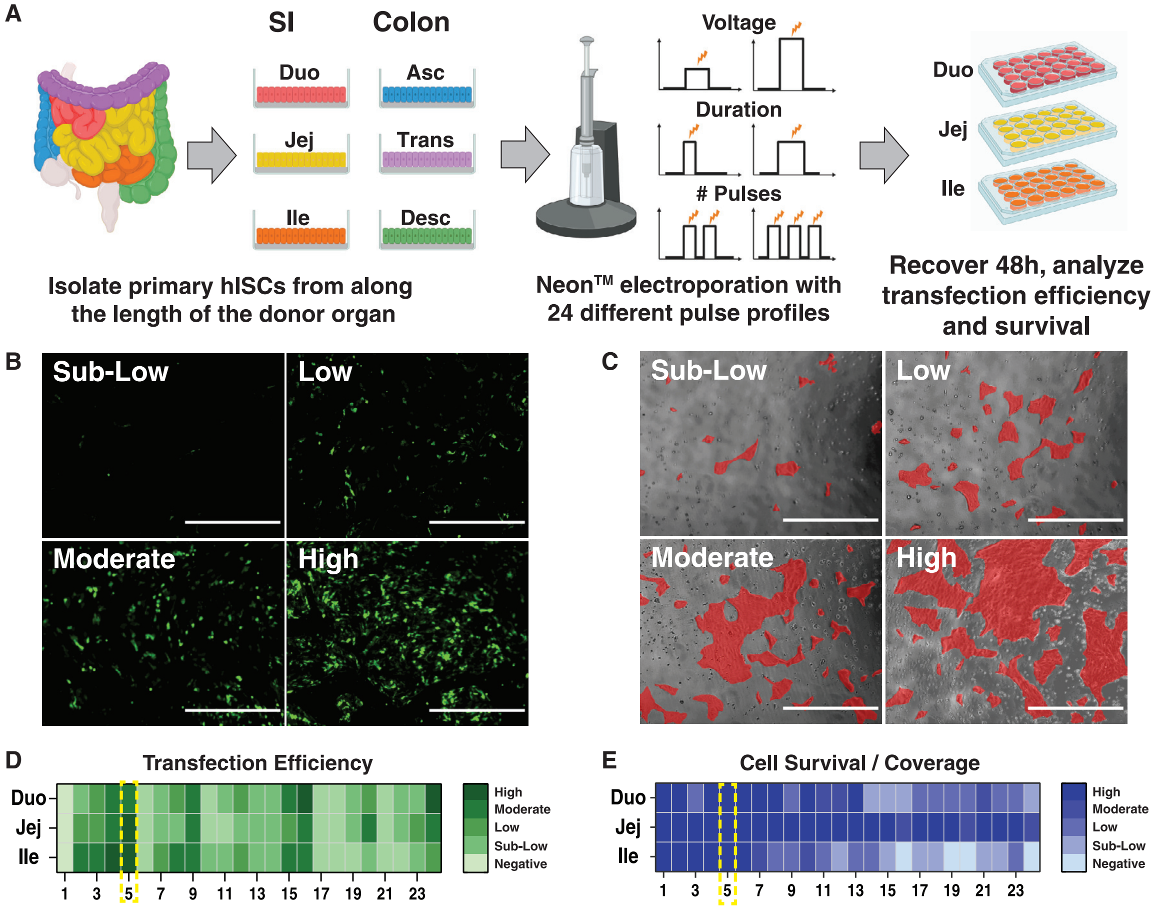

Figure 1. A single high-voltage pulse efficiently transfects hISCs

(A) Workflow for hISC sourcing and electroporation optimization. Colors represent different isolated regions of the intestinal tract.

(B) Representative images of cell densities used to bin transfection efficiency: negative (no visible GFP, not pictured), sub-low (<10% of cells expressing GFP), low (10%–30%), moderate (30%–50%), and high (>50%); Scale bars, 2,000 μm.

(C) Representative images of cell densities used to bin cell survival and coverage. For visualization, cell patches are overlaid in red. Survival was estimated as percent coverage compared to an untransfected control: negative (no surviving cells, not pictured), sub-low (<10% coverage compared to negative control), low (10%–30%), moderate (30%–50%), and high (>50%); Scale bars, 2,000 μm.

(D) Heatmap of transfection efficiencies from 24 tested electroporation parameters with optimal parameters highlighted.

(E) Heatmap of cell survival/coverage from 24 tested electroporation parameters with optimal parameters highlighted. See also Table S1.

PiggyBac-integration events, we generated four PiggyBac donor vectors, each containing an antibiotic selection marker and a different constitutively expressed fluorescent reporter gene (mTagBFP2, H2B-EGFP, mApple, and iRFP670) that exhibit a very low spectral overlap (Figure 4A). To determine the efficiency of integration, fluorescent reporter gene plasmids were individually transfected into dissociated hISCs. Stable integration was quantified after antibiotic selection by counting well-isolated fluorescent colonies. Six transfections were performed in parallel in jejunal and descending colon monolayers, generating approximately 1,700 antibiotic-resistant fluorescent colonies in approximately 3 h. The integration incidence per 10^5 transfected cells after experimental normalization was 176 ± 36 colonies (0.18%) in jejunum and 107 ± 15 (0.11%) colonies in descending colon (Figure 4B), approximately three- to four-fold higher than reported PiggyBac integration efficiency in 3D organoids (Fujii et al., 2015). Thus, with a typical transfection input of 10^6 cells, a mean transfection efficiency of 43.8%, and 100–150 colonies per 10^5 transfected cells, a single PiggyBac transfection is expected to yield 450–750 colonies on average.

Some experimental designs require the integration of multiple transgenes into the same hISC line. To quantify integration of multiple plasmids in the same cell, equal molar ratios of each fluorescent reporter plasmid were

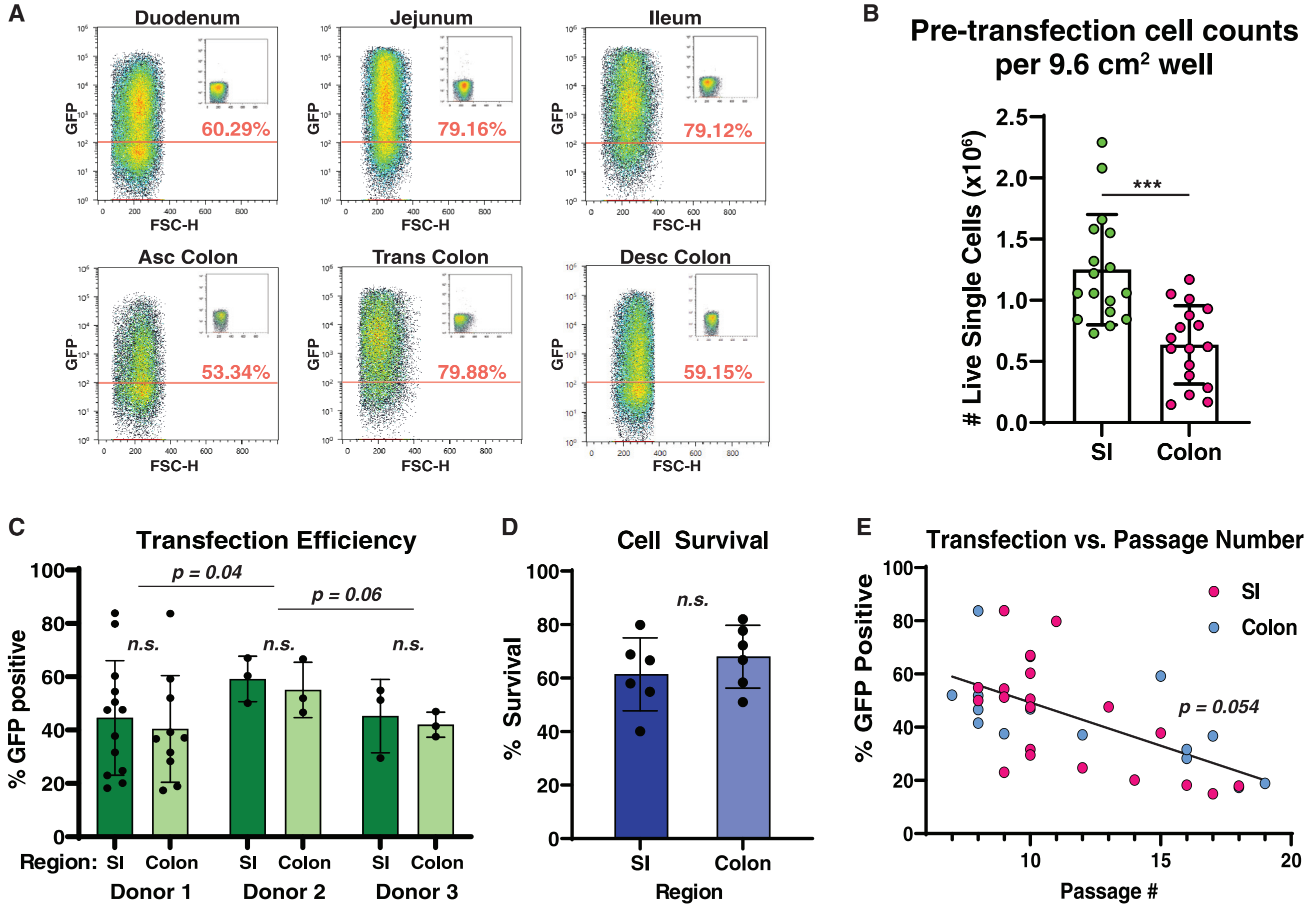

Figure 2. Optimized parameters are robust regardless of source region and donor

(A) Flow cytometry density plots of highest transfection efficiency observed for each of six intestinal segments using optimized parameters (1,700 V, 20 ms, 1 pulse). Insets show matched untransfected controls.

(B) Summary of live (Trypan Blue negative) single cells obtained from each well of a six-well culture plate. $p < 0.001$, n = 17 independent experiments per organ, each data point represents the average of three to six dissociated wells. Mean ± SEM.

(C) Summary of flow cytometry-measured transfection efficiencies using chosen parameters, data points represent independent experiments. Mean ± SEM.

(D) Summary of hemocytometer-measured cell survival after transfection as a percentage of cell numbers in matched untransfected controls; p = 0.38, n = 6 independent experiments per organ. Mean ± SEM.

(E) Transfection efficiency (from Figure 2C), plotted as a function of passage number with line of best fit. See also Table S2.

mixed together and electroporated into hISCs from the jejunum and descending colon. Drug-resistant colonies were evaluated for expression of the four different fluorescent reporter genes and the number of different colors expressed in each colony was quantified under fluorescence microscopy (Figure 4C). Jejunal hISCs had single plasmid integration in approximately 45% of colonies, two or three different plasmids integrated in approximately 20%, and four plasmids integrated in approximately 10% of colonies (Figure 4D). Descending colon hISCs had single plasmid integration in approximately 35% of colonies, two or three different plasmids integrated in approximately 15%–20% of clones, and all four plasmids integrated in approximately 30% of colonies (Figure 4D). These findings indicate multiple constructs can be efficiently integrated into the genome of the same cell.

Optimized electroporation parameters are efficient at transfecting CRIPSR/Cas9 RNP complexes

An alternative approach to random transgene integration is loci-directed integration by CRISPR-based gene editing, which is useful for gain- and loss-of-function studies and generating lineage reporter gene expression driven by endogenous regulatory elements (Cong et al., 2013; Mali et al., 2013). Transfecting ribonucleoprotein (RNP) complexes instead of Cas9-expressing plasmids and guide

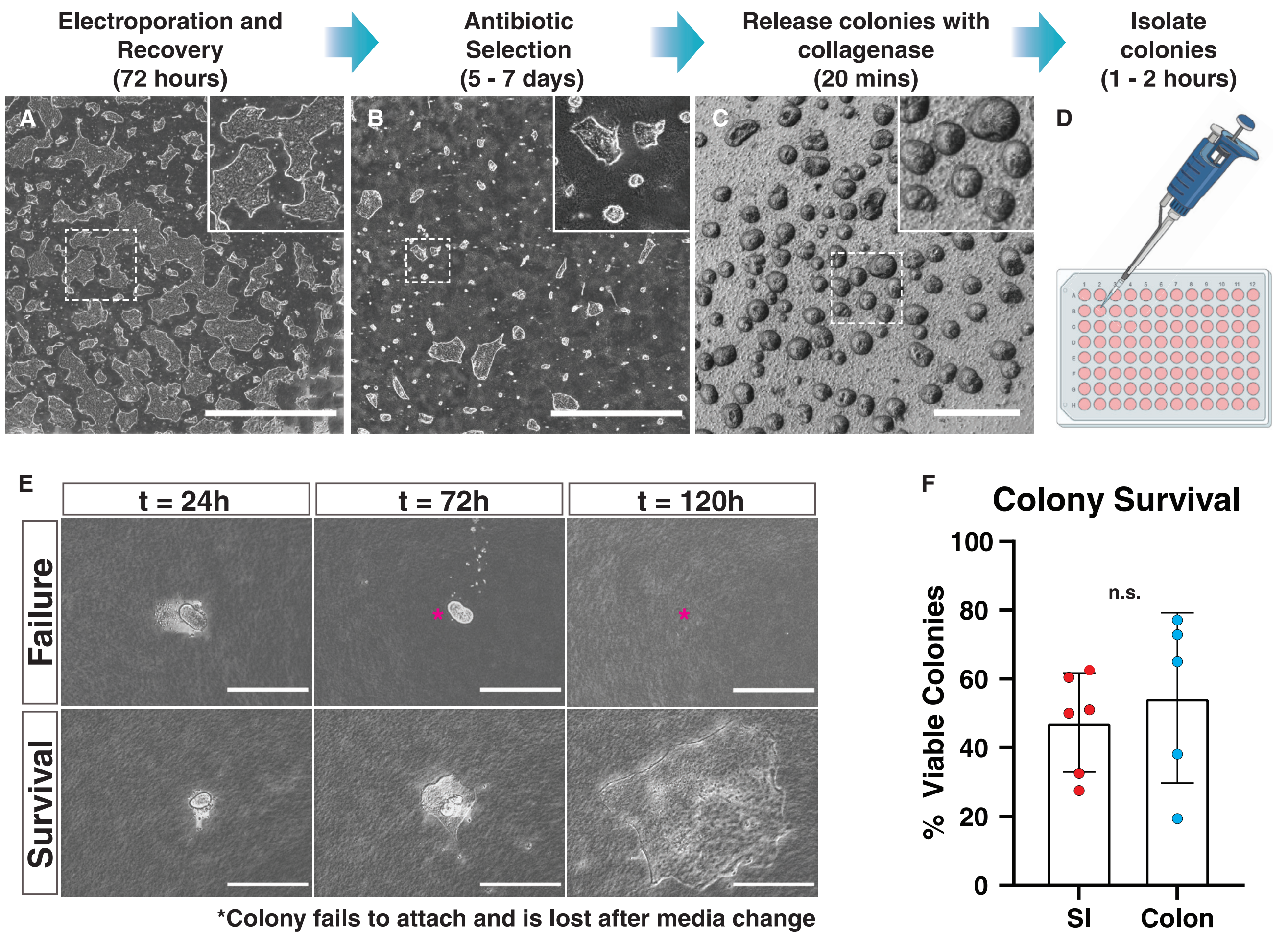

Figure 3. Colony isolation from 2D collagen monolayers is high throughput

(A–D) Overview of transfection and colony isolation workflow with representative images from each step. Scale bars (A–B), 4,000 μm; (C) 400 μm.

(E) Representative time course of colony attachment and growth after isolation. Asterisk indicates original colony position of non-adherent colony. Scale bars, 200 μm.

(F) Quantification of colony survival following isolation; $p = 0.43$, $n > 4$ independent experiments per organ. Mean ± SEM.

RNAs (gRNAs) independently is attractive for delivering CRISPR reagents owing to the decreased potential for off-target cleavage or integration (Liang et al., 2015). As a candidate locus to test RNP targeting efficiency in hISCs, *OLFM4* was chosen for its robust expression in small intestinal hISCs, as demonstrated by *in situ* hybridization, immunostaining, and single-cell RNA sequencing (Burclaff and Bliton, 2022; Busslinger et al., 2021; van der Flier et al., 2009; Gersemann et al., 2012).

A gRNA targeting the 3′ UTR of *OLFM4* was designed using CCTop (which identifies and ranks gRNAs according to predicted off-target activity) (Stemmer et al., 2015) and selected to minimize risk for off-target cleavage (Table S3). Assembled Cas9/gRNA RNPs were electroporated into jejunal and descending colon hISCs. Following recovery, bulk DNA was extracted from transfected cells and the *OLFM4* locus was sequenced (Table S4). TIDE analysis, which detects and quantifies insertion/deletion (indel) mutations by sequencing (Brinkman et al., 2014), showed 34.8% ± 13.4% indel formation efficiency at this locus, with no significant difference between SI and colon hISCs (Figure 5A). These findings show that RNPs are an efficient alternative to plasmid-dependent Cas9 expression for gene editing using these methods.

RNP complexes were next used to generate an OLFM4-emGFP reporter line in duodenal hISCs by co-electroporating a donor plasmid containing an IRES-emGFP cassette flanked by approximately 1 kb of wild-type sequence to facilitate integration through homology-directed repair (HDR) (Figure 5B). By integrating into the 3′ UTR of the

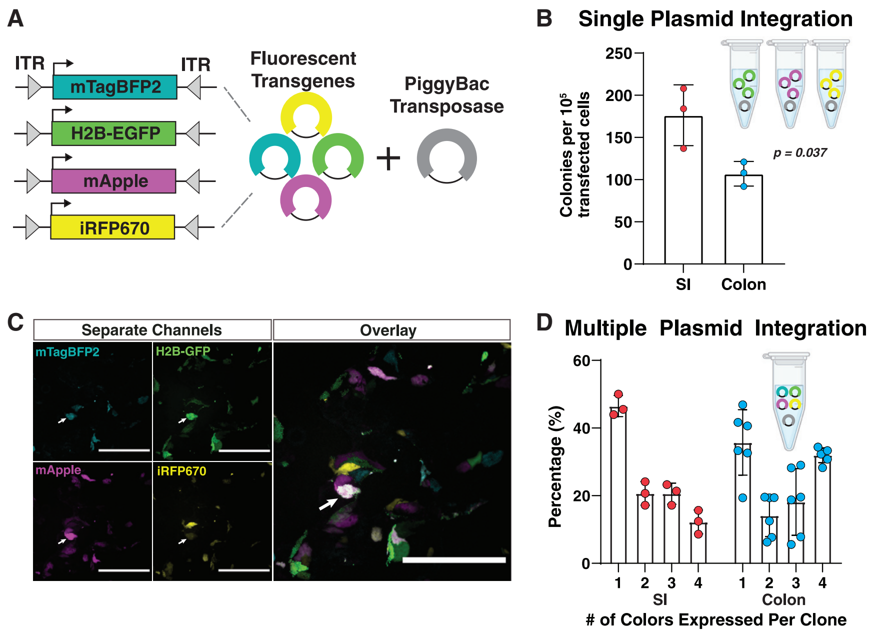

Figure 4. PiggyBac transfection yields robust single- and multiple-plasmid integration

(A) Schematic of plasmids with constitutively expressed fluorophores flanked by transposase-binding inverted tandem repeats.

(B) Quantification of colony numbers after antibiotic selection for PiggyBac integration, normalized by FACS-measured transfection efficiency; $p = 0.037$, $n = 3$ parallel transfections of different plasmids for each organ. Mean ± SEM.

(C) Representative images of hISCs transfected with the four fluorescent reporter plasmids (Figure 4A). Arrow indicates a colony that has integrated all four reporter plasmids; Scale bars 200 μm.

(D) Quantification of multiple integration events observed in stably transfected colonies as represented in Figure 4C. Each data point represents the percentage of a clone type in a different field of view from a single experiment. Mean ± SEM.

OLFM4 locus, the IRES-emGFP cassette reports the presence of *OLFM4* mRNA without disrupting the *OLFM4* coding sequence (Figure 5B). RNP complexes were electroporated into approximately 10^6 hISCs, and visual inspection by brightfield microscopy estimated approximately 367 antibiotic resistant clones survived selection. Fluorescent microscopy confirmed emGFP expression in 45% of colonies (Figures 5C and 5D). Forty-eight clones were isolated and expanded in monolayer culture as described above. Five emGFP$^+$ clones were evaluated by PCR, with all exhibiting proper integration of the IRES-emGFP cassette in the 3′ UTR of *OLFM4* (Figure S1A). Two clones (D7 and C9) were chosen for further characterization, validating the absence of off-target mutations (Figure S1B) and karyotypic normality (Figure S1C). These data demonstrate efficient HDR-mediated gene integration and robust reporter expression from the *OLFM4* locus, highlighting its utility as an hISC reporter gene.

Olfm4-emGFP hISCs generated by Cas9-mediated transgenesis display compartmentalized emGFP expression on a planar crypt-microarray (PCM) device

Since multiple colonies showed OLFM4-emGFP expression and PCR-validated insertion into the 3′ UTR of the *OLFM4* locus, we sought to validate that the reporter accurately reflected endogenous *OLFM4* expression. To do this, a recently developed PCM 2D crypt array system was used (Kim et al., 2018). The PCM is a microfabricated culture surface that replaces the fully permeable membrane of a Transwell insert with an impermeable membrane with permeable holes arranged in a grid-like pattern across its surface (Figure 6A). The dimensions of the permeable regions mimic the scale and distance between crypts *in vivo*. Tissue zonation, defined as compartmentalization of proliferative and differentiated zones, is forced by applying hISC media to the basal reservoir and differentiation media to the apical reservoir (Figure 6A). The hISCs are only maintained over permeable holes in the culture surface, which provide access to the hISC media (basal reservoir), while impermeable zones lacking access to the hISC media differentiate.

The OLFM4-emGFP hISC reporter lines were applied to PCMs and the cells were first allowed to expand with hISC media on both the apical and basal sides of the device (Figure 6B). After the hISCs reached confluence, the hISC media in the apical reservoir was replaced with differentiation media lacking hISC factors creating a steep gradient of hISC maintenance factors over the permeable crypt regions (Figure 6B). After 4 days, the tissue compartmentalized into OLFM4-emGFP positive and negative zones that persisted for at least 2 days, which was the latest time point tested (Figures 6B, 6C, 6D, and S2A). OLFM4-emGFP was strongly expressed near permeable zones, peaking 20 μm outside of the permeable region, but not in intervening regions (Figure 6C). Immunostaining for the general proliferation marker (KI67) showed proliferative cells (i.e., hISC/progenitors) (Barker et al., 2007) localized primarily around permeable crypt zones (Figure 6D). By contrast, Cytokeratin 20 (KRT20), a pan-marker of differentiated intestinal epithelial cells (Merlos-Suárez et al., 2011; Moll et al., 1990), localized primarily to intervening impermeable

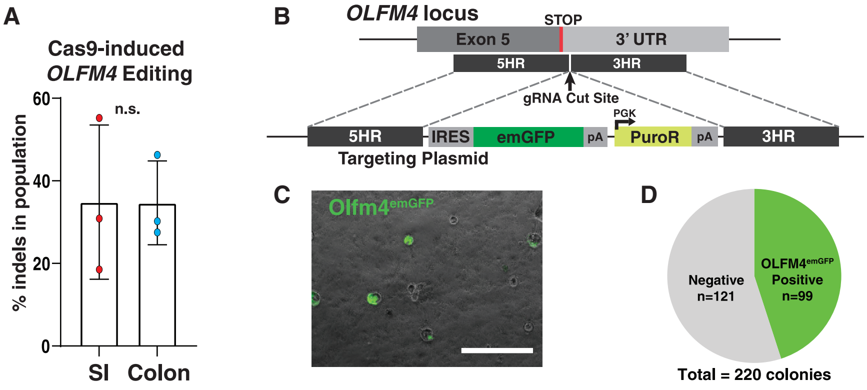

Figure 5. Cas9 RNP transfection produces efficient HDR at the *OLFM4* locus

(A) Percentage of Cas9-induced indels at the *OLFM4* gRNA cleavage site, measured by TIDE analysis of bulk DNA; $p > 0.99$, $n = 3$ independent experiments per organ. Mean ± SEM.
(B) Schematic of the *OLFM4* insertion site and plasmid used for targeting, showing IRES-emGFP insertion in the *OLFM4* 3′ UTR along with a constitutively expressed puromycin resistance gene.
(C) Representative image of OLFM4^{emGFP} colonies following antibiotic selection. Scale bar, 1,000 μm.
(D) Quantification of emGFP + colonies compared with total colony numbers after antibiotic selection. See also Tables S3, S4, and Figure S1.

regions (Figure 6D). Confocal microscopy demonstrated robust co-localization of OLFM4-emGFP and KI67 over permeable crypt zones and visibly low or no transgene expression in intervening zones (Figure 6D). This demonstrates that the OLFM4-emGFP reporter cells retain hISC characteristics in that they respond to self-renewal signaling and can differentiate into post-mitotic lineages. Additionally, these findings suggest the OLFM4-emGFP reporter can distinguish between functional ISCs and differentiated cells in culture.

Olfm4-emGFP cells mimic endogenous *OLFM4* expression and allow for isolation of *LGR5*^{high}/ *KRT20*^{low} cells with high organoid-forming potential

Cells zonated on the PCM demonstrated a range of OLFM4-emGFP expression levels via flow cytometry, classified as negative, low (bottom 50% of EGFP + cells), and high (top 50% of GFP + cells). Gates for fluorescence-activated cell sorting (FACS) were defined using these three categories (Figures 7A and S2B). Interestingly, the forward scatter width (FSC-W) parameter indicated a separate density of cells within the OLFM4-emGFP^{high} population, suggesting a population of cells with a smaller diameter than the main distribution of cells (High^{Sm}) (Figure 7A). Smaller diameter was confirmed by measuring sorted cells (Figure 7B), and additional FACS gates were included to compare the smaller OLFM4-emGFP^{high} cells with the larger OLFM4-emGFP^{high} cells based on the FSC-W parameter (High^{Lg}, Figure 7A).

To verify that the OLFM4-emGFP signal reflected endogenous *OLFM4* expression, cells from each gate were FACS isolated and cDNA was generated for qPCR (Figures 7C and S2C). OLFM4-emGFP transgene expression was consistent with endogenous *OLFM4* expression. Markers of ISCs and proliferation, *LGR5* and *KI67*, tracked with increasing *OLFM4* and the OLFM4-emGFP transgene. Conversely, the pan-differentiation marker *KRT20* negatively correlated with ISC and proliferation marker genes. Interestingly, the smaller OLFM4-emGFP^{high} population (High^{Sm}) expressed significantly higher levels of hISC marker genes than the larger OLFM4-emGFP^{high} population (High^{Lg}). These results show that cells zonated on PCMs demonstrate a range of *OLFM4* mRNA expression and suggest functional differences associated with OLFM4-emGFP expression and cell size.

Organoid-forming capacity from single cells is a common *in vitro* test of functional stemness (Sato et al., 2009). To investigate the relationship between OLFM4-emGFP expression and stem cell function, single cells from each OLFM4-emGFP population were FACS-sorted into Matrigel and assayed for their ability to form organoids. Less than 1% of cells from the GFP-negative and GFP-low gates formed organoids, while 2.5% of High^{Lg} cells and 7.7% of High^{Sm} cells formed organoids ($p < 0.001$) (Figure 7D). This demonstrates a strong positive correlation between OLFM4-emGFP expression and organoid-forming capacity and indicates that smaller OLFM4-EGFP^{high} cells have higher hISC activity in culture.

DISCUSSION

Here, we present new methods for generating and isolating transgenic small intestinal and colonic hISCs grown as 2D monolayers. We optimize electroporation parameters and demonstrate robust transfection of hISCs isolated from six regions of the intestinal tract with a high incidence of cell survival. We show these parameters efficiently transfect both DNA (PiggyBac plasmids) and protein (Cas9 RNP complexes) and demonstrate utility by

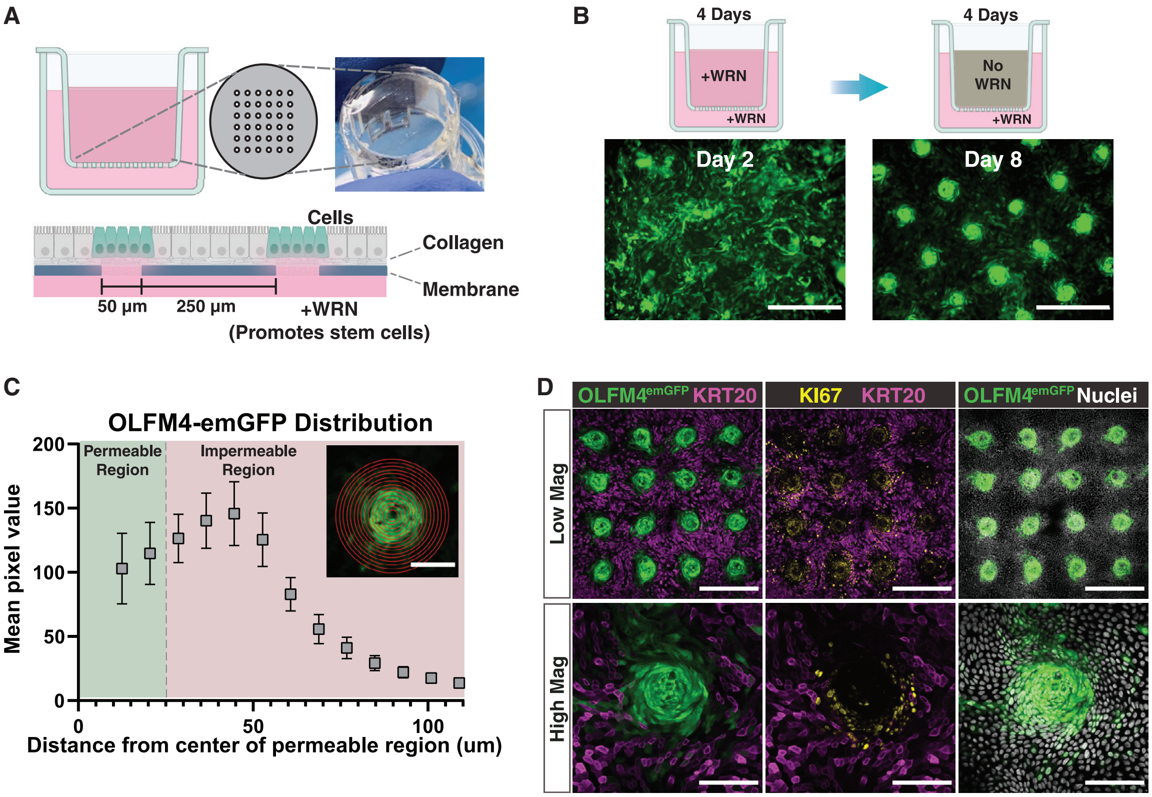

Figure 6. An OLFM4^{emGFP} hISC line on engineered 2D crypt arrays reports proliferative high-Wnt zones

(A) Planar crypt-microarray (PCM) used for hISC zonation. An array of microholes in an impermeable membrane restricts access to the hISC media reservoir, promoting stem cell maintenance only over the holes.

(B) Overview of growth and zonation protocol for hISCs grown on PCMs. Cells are grown 4 days with hISC media in both compartments followed by 4 days with differentiation media in the apical compartment. Scale bars, 200 μm.

(C) Distribution of OLFM4-emGFP signal measured by averaging the pixel intensity on concentric circles overlaid on 16 confocal images of different microholes. Inset is a representative image overlaid with the concentric circles used. Scale bar, 100 μm. Mean ± SEM.

(D) Confocal microscopy of immunostained OLFM4^{emGFP} hISCs grown on PCMs. Scale bars: Low Mag, 400 μm; High Mag, 100 μm. See also Figure S2.

generating and validating a novel and robust OLFM4-emGFP hISC reporter line.

Our data demonstrate a broad range of transfection efficiencies ranging from 15% to 80%, despite using optimized electroporation parameters. While the exact mechanisms mediating this variability are not well understood, several factors may contribute. Cell passage number has been shown in other reports to impact transfection efficiencies (de Carvalho et al., 2018; McCoy et al., 2010). Our findings are consistent with this as there was a negative correlation between transfection efficiency and increasing cell passage number. Proliferative state and cell density can also affect transfection efficiency (Prasanna and Panda, 1997). We did not explore the impact of these parameters in this study as transfection efficiencies were sufficiency high for most applications. hISC media components that impact proliferation and stem cell states are numerous, highly variable, and could contribute the inconsistencies in transfection efficiencies, transgenesis, and gene editing (Hinman et al., 2021). Standardization of critical hISC maintenance factors (i.e., WNT3A, NOGGIN, R-SPONDIN, *etc*) produced by cell lines, and FBS as an essential culture component (Wang et al., 2017a), is very challenging as these reagents are composed of unknown components, variable growth factor concentrations, and heterogeneous production practices (Honn et al., 1975). Thus, cell passage number, proliferative state, density of hISCs, and media standardization could be further optimized and should be considered during experimental design when very high transfections efficiencies are required.

A major challenge in genetic engineering is isolating colonies that are truly clonal, that is, colonies that come from a single gene-edited cell. The likelihood of obtaining clonal populations is significantly improved by a culture system

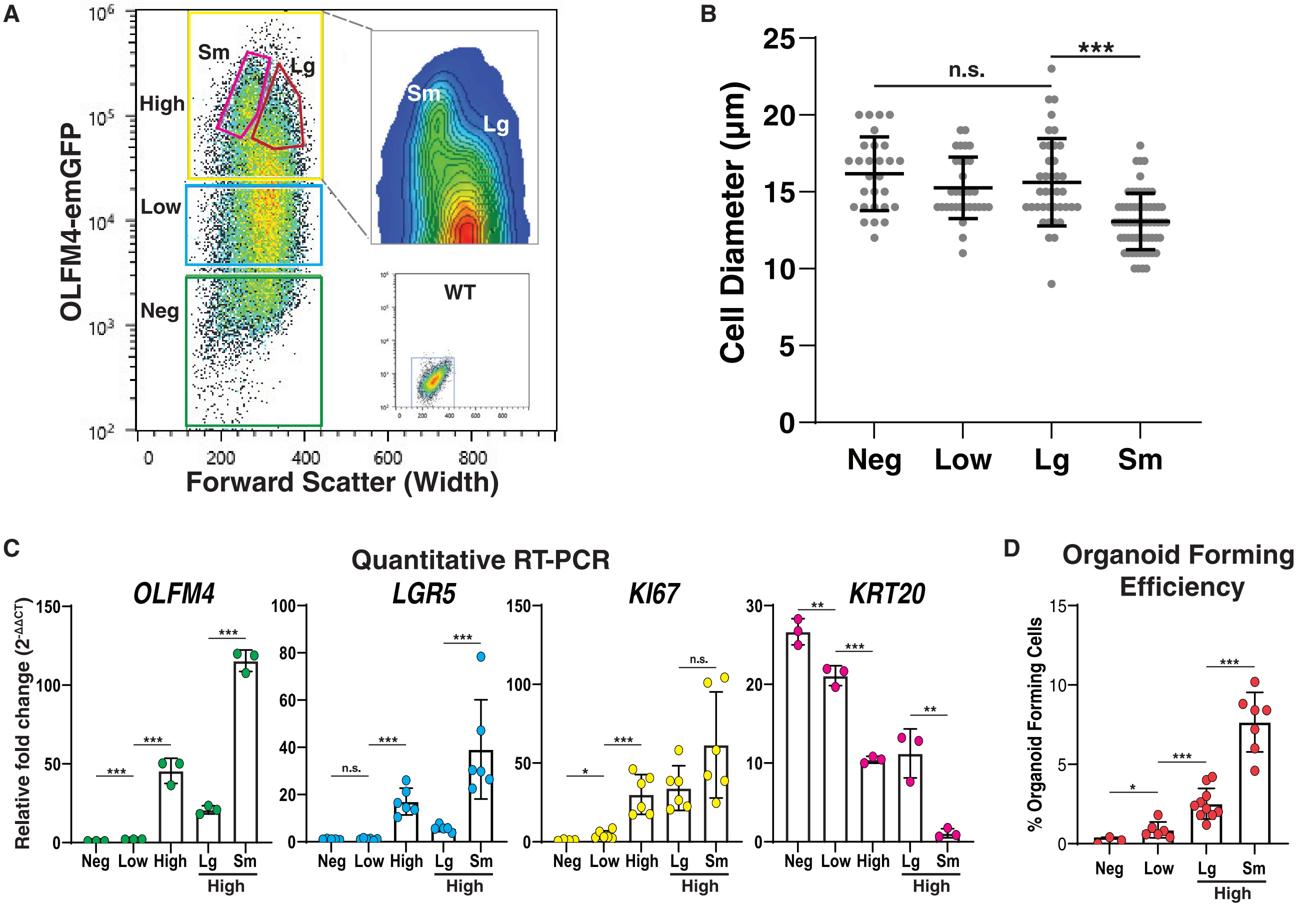

Figure 7. Small *OLFM4*^{High} cells demonstrate increased organoid forming efficiency *in vitro*

(A) FACS density plot of OLFM4^{emGFP} cells isolated from a zonated PCM, overlaid with gates used for sorting. Insets show WT negative control and a contour plot of the High gate.

(B) Microscopy-measured cell diameters for cells sorted from each FACS gate in Figure 7A n > 27 cells per gate. ***p < 0.001. Mean ± SEM.

(C) Relative gene expression measured by qRT-PCR on FACS-sorted cells obtained from each gate in Figure 7A. Data normalized to Neg for *OFLM4*, *LGR5*, and *KI67*, and to High^{sm} for *KRT20*; *p < 0.05, **p < 0.01, ***p < 0.001. Mean ± SEM.

(D) Quantification of organoid formation by FACS-isolated single cells obtained from zonated PCMs; *p < 0.05, ***p < 0.001, each data point represents the percentage of cells that formed organoids following seeding of 500 single cells into a separate Matrigel patty. Mean ± SEM. See also Figure S2.

the facilitates spatial separation of colonies following antibiotic selection. Experimental design to achieve well-isolated clones is dependent on transfection efficiency, payload integration efficiency, and the number of transfected cells. For instances, the high efficiency and integration of PiggyBac-mediated transgenesis in our study yielded many antibiotic-resistant clones that merged as they grew. By contrast, the lower efficiency of HDR yielded far less antibiotic-resistant clones with more spatial separation. Thus, when transfection efficiencies are high, serial dilution of transfected hISCs is recommended to limit the merging of clones with independent transgenesis or gene-editing events.

Transgenesis and gene editing using 2D cultures of hISCs has a number of advantages including improved throughput and substantial cost savings over transgenesis in 3D organoid cultures. The familiarity of 2D culture in laboratory settings reduces training time and failures inherent with more complex 3D hydrogel culture systems. When using hISCs expanded in 3D hydrogels, many wells of Matrigel patties are required to generate the numbers of hISCs for transfection. For example, one highly cited report recommends up to 24 patties to obtain 5×10^5 live single cells for a single targeted knock-in experiment (Fujii et al., 2015). By contrast, our findings show one well of a conventional six-well plate yields an average of 0.64×10^6 to

1.25 × 10^6 live single cells (Figure 2B), comparable with the yield of 30–60 Matrigel patties. The culture-well form factor and the low cost of collagen hydrogels used in 2D hISC expansion significantly reduces the cost of transgenesis projects. Based on the above numbers and current prices we estimate that 2D hISC expansion methods reduces costs to produce equivalent numbers of hISCs by 16- to 32-fold.

Transgenic hISC lines that report cell lineage states are powerful tools to investigate various aspects of gut epithelial biology, and delineate important differences and confirm findings in murine models of human physiology and disease. In this study we generated and characterized an OLFM4-emGFP hISC line. *OLFM4* has been shown by *in situ* hybridization and scRNA sequencing to be robustly expressed by human crypt-base columnar cells (CBCs), with gradually decreasing expression up the crypt axis (Burclaff and Bliton, 2022; Busslinger et al., 2021; van der Flier et al., 2009). While *OLFM4* demonstrates broader crypt expression than *LGR5*, single-cell RNAseq data show that the highest *OLFM4* expression overlaps with the highest *LGR5* expression (Burclaff and Bliton, 2022). Using engineered gut epithelial tissue constructs generated on planar-crypt microarrays (PCMs), our data revealed a robust gradient of OLFM4-emGFP expression levels with a direct correlation with endogenous *OLFM4*, *LGR5*, and *KI67* levels, and highest hISC organoid forming capacity correlated with highest OLFM4-emGFP expression and smaller cell diameter. While the functional implications of cell size are not well understood, small cell size has been correlated with high stem cell function in several stem cell types (Kucia et al., 2006; Paiva et al., 2006). Together these results show that OLFM4-emGFP reporter hISCs have functional ISC activity, respond to hISC maintenance signals, and differentiate when removed from these signals.

Transgenesis and gene editing in hISCs cultured as 2D monolayers holds enormous potential for studying intestinal biology and disease in physiologically relevant culture systems. Additionally, targeted genetic manipulation in hISCs could be leveraged for high-throughput drug screening and development of cell-based therapies for a number of human health conditions. Our study provides robust, reproducible, and easily scalable methods that are essential for progress toward these goals.

EXPERIMENTAL PROCEDURES

hISC media and plate preparation

Maintenance media (MM) for hISC culture was prepared as previously described (Hinman et al., 2021). Briefly, L cells expressing transgenic WNT3A, NOGGIN, and RSPONDIN3 (ATCC CRL-3276) were cultured in L-WRN medium (Table S5). Media was collected and replaced every 24h for 12 days. Collected conditioned medium (CM) was then pooled, filtered, and frozen into aliquots stored at −80°C. Thawed CM aliquots were mixed 1:1 with basal medium (Table S5). For plating of primary crypts and after passaging, MM was supplemented with 10 μM Y27632 (MM + Y, Selleck Chemical S6390). Collagen-coated plates for monolayer culture were prepared as previously described (Wang et al., 2017a).

Tissue procurement and dissection

Intestinal tissue was obtained postmortem from HonorBridge from three organ donors with no known history of intestinal disease and negative for an infectious disease panel (Table S2). Following trimming of adipose tissue, the first 20 cm of the SI was isolated. A 9-cm^2 tissue sample was taken from the center of this piece (duodenum). The remaining SI was divided in half, and tissue samples were taken from the middle of each half (jejunum and ileum). The colon was divided into three equal length pieces, and tissue samples were taken from the center of each piece (ascending, transverse, and descending colon). Tissue samples were stored in Advanced DMEM/F12 + 10 μM Y27632 and 200 μg/mL Primocin during dissection, immediately followed by crypt isolation.

Crypt isolation and hISC expansion

After dissection, tissue sections were incubated in cold PBS +10 mM N-acetylcysteine with gentle agitation for 15 min. They were then rinsed briefly in isolation buffer (IB, Table S5), transferred to IB + 2 mM EDTA (Corning 46-034-Cl) + 0.5 mM DTT (ThermoFisher Scientific BP172-5), and incubated for 30 min with gentle agitation. Tubes were shaken vigorously for 2 min, after which sections were transferred to new tubes of IB + EDTA + DTT and rocked gently for 10 min. This process of shaking, transfer, and rocking was repeated six times. Buffer aliquots were analyzed under widefield microscopy to quantify villi and crypt numbers, and crypt-enriched samples were pooled. Crypt numbers were counted and seeded on collagen plates in hISC expansion media with Y27632 (MM + Y, described above), 200 μg/mL Primocin, 200 μg/mL Gentamycin (Sigma Aldrich G1914), and 0.5 μg/mL amphotericin B (Sigma-Aldrich A2942) (+antibiotics) at 5,000 crypts per well of a six-well plate. Plates were incubated at 37°C and media was changed daily with fresh MM + antibiotics.

hISC monolayer culture and passaging

Human ISCs were cultured at 37°C, 5% CO_2 in 3 mL of MM as described above, replacing the media every 2 days. Cells were passaged 1:3 every 4–7 days based on cell density, with a pre-passage confluency of greater than 60%. We added 10 μM Y27632 to each well at least 1 h before passaging. To passage, 1 mL of media was removed from each well into a conical tube and the remaining media aspirated. Collagen patties were dislodged with a pipet tip and transferred to the tube. 100 μL of 5,000 U/mL collagenase IV (ThermoFisher Scientific LS004189) in HBSS (Gibco 14175-095) was added to each tube and the collagen patties were broken up by pipetting with a 5-mL serological pipet. This solution was incubated at 37°C for 20 min, mixing every 5 min, then pelleted at 800 × g for 3 min. Supernatant was aspirated and the cell pellet resuspended in 5 mL pre-warmed 1× PBS +10 μM Y27632. PBS suspensions were incubated at 37°C for 5 min, then pelleted at 800 × g for 3 min. PBS was aspirated and cell pellets were resuspended in 200 μL TrypLE Express (Gibco 12605-010) and

incubated at 37°C for 5 min. TrypLE solutions were pipetted approximately 15 times with a P1000 pipet tip to dissociate cells, then neutralized with 2 mL MM and pelleted again at 800 × g for 3 min. Supernatant was aspirated and the cells were resuspended in 9 mL MM + Y per dissociated well and plated onto three new pre-rinsed collagen-coated wells (3 mL/well).

OLFM4 targeting plasmid generation and gRNA selection

OLFM4 homology regions were amplified from donor gDNA with CloneAmp HiFi PCR Premix (Takara 639298) using primers KB39 + KB40 and KB69 + KB38 (Table S4), which included 20 bp of overlapping homology to plasmid backbone elements, and purified on a silica minicolumn. IRES-emGFP and vector backbone were isolated from a pre-existing plasmid by restriction digestion and assembled with PCR-amplified homology arms using an In-Fusion HD Cloning Kit (Takara 638920). The final products were verified by enzymatic digestion and sequencing. The *OLFM4* gRNA was designed using CCTop (Stemmer et al., 2015) and were selected based on location in the proximal 3′ UTR and low probability of off-target cleavage (Table S3).

Preparation of transfection reagents

Super PiggyBac Transposase Expression Vector (System Biosciences, PB210PA-1) was added at 5 ng/μL in transfection mix. All other plasmids were prepared from bacterial stocks using a QIAGEN HiSpeed Maxi kit (QIAGEN 12662) and concentrated to 1–2 μg/μL in TE Buffer. pMax-GFP plasmid was added at 100 ng/μL of transfection mix. All other plasmids were added at 50 ng/μL. For gRNA and Cas9 transfections, crRNA, and tracrRNA oligos were purchased from IDT (Alt-R® CRISPR-Cas9 system) and resuspended at 100 pmol/μL in nuclease-free dH2O (Corning 46-000-Cl). We combined 2 μL (200 pmol) each of gRNA and tracRNA with 2 μL of 5X annealing buffer (ThermoFisher Scientific 100061876) and 4 μL nuclease-free dH2O and annealed in a thermocycler (95°C – 5 min, 95°C to 78°C at −2°C/s, 78°C – 10 min, 78°C to 25°C at −0.1°C/s, 25°C – 5 min), then immediately transferred to ice. We added 3 μL (60 pmol) of this mix to 2 μL TrueCut Cas9v2 (approximately 60 pmol, ThermoFisher Scientific A36498), incubated at room temperature for 15 min to promote formation of RNP complexes, then stored on ice until use.

Single-cell dissociation for transfection and flow cytometry

To improve cell dissociation for transfection, several changes were made to the passaging protocol described above. The length of the 37°C PBS incubation was extended to 10 min, with gentle mixing after the first 5 min. Next, 20 μL of 5,000 U/mL collagenase IV was added to the 200 μL TrypLE for dissociation. Following the 5 min incubation in TrypLE + collagenase, cells were first dissociated by pipetting approximately 15 times with a P1000 pipet tip as above, then further dissociated by gently pipetting with a 28G insulin syringe (BD Biosciences 329,424) 5–7 times. Dissociated TrypLE solutions were neutralized, centrifuged, and aspirated normally, but were resuspended in 1 mL 1× room temperature PBS. We removed 10 μL of this cell suspension and it mixed 1:1 with Trypan blue and cell numbers were quantified with a hemocytometer. PBS solutions were again pelleted at 800 × g for 3 min, the supernatant aspirated, and the cell pellet resuspended in cold Neon Buffer R (ThermoFisher MPK10096) at a concentration of 5,000–20,000 cells/μL. Flow cytometry was performed using a Sony SH800ZF cell sorter (SN 1500005).

Electroporation and antibiotic selection

For electroporation, cells were dissociated to singlets as described above. For testing of electroporation conditions, cells were resuspended at a concentration of 20,000 cells/μL in Neon Buffer R, and pMax-GFP was added at 100 ng/μL of buffer. After electroporation with a 10 μL Neon tip (ThermoFisher MPK1096), cells were seeded onto a single well of a collagen-coated 24-well plate with MM + Y and allowed to recover overnight at 37°C. Media were replaced after 24 h with MM, and cells were analyzed for transfection and survival after 72 h. After optimization, all other transfections were performed using the Neon Electroporator 100 μL tips (ThermoFisher Scientific MPK10096) with preset #5 (1,700 V, 1 pulse, 20 ms), electroporating 6,000–12,000 cells/μL and plating onto a single collagen-coated well of a six-well plate. Media was changed after 24 h with MM. For transfections requiring selection, media were changed with MM + puromycin (2 μg/mL, Sigma P8833) after 72 h and every other day thereafter for 7 days. To account for experimental variability in single plasmid integration rates, the transfection efficiency for each plasmid was normalized to the efficiency of a control transfection using a constitutively expressing EGFP plasmid (Figure 4B).

Colony isolation and expansion

To isolate transgenic colonies after antibiotic selection, 300 μL of 5,000 U/mL collagenase IV was added to each well and incubated at 37°C for 20 min. Supernatant and dislodged colonies were collected and pelleted at 600 × g. Media was aspirated and the cells gently resuspended in 10 mL PBS +10 μM Y27632 (PBS + Y). Cells were re-pelleted, and suspended in a new aliquot of 15 mL PBS + Y and added to a 10-cm tissue culture dish. This dish was transferred to an inverted light microscope, and colonies were manually pipetted into a collagen-coated 96-well culture plate containing 100 μL/well MM + Y. After isolation, colony media was changed every other day with MM + Y. Upon reaching confluency or after 2 weeks, colonies were serially passaged to collagen-coated 48-well, 12-well, and 6-well culture plates.

PCM fabrication

The PCMs were fabricated as previously described (Kim et al., 2018). Briefly, an array of 20 × 20 microholes (50 μm in diameter) were micropatterned onto a thin, impermeable 1002F-10 photoresist film using a photomask (Front Range Photomask) and UV photolithography (Pai et al., 2007). The micropatterned film was then mounted onto a bottomless 12-well Transwell insert using 3M double-sided medical tape and coated with 200 μL of 1 mg/mL neutralized rat tail collagen I (Corning 354,236). Collagen-coated PCMs were incubated in a humidified 37°C chamber for 1 h to promote formation of a uniform gel, dried overnight

in a 40°C oven, then stored at room temperature. Before seeding cells, PCM Transwells were sterilized with ethanol, then rehydrated with 50 μg/mL rat tail collagen I in PBS overnight at 37°C.

Culturing hISCs on PCM devices and immunostaining

Confluent six-well monolayers of OLFM4-emGFP cells were dissociated as previously described, passaging one well of a six-well plate to three PCM devices in MM + Y, adding 500 μL medium with dissociated cells to the upper compartment and 1.5 mL medium (without cells) to the lower compartment. Media was changed after 48 h with fresh MM + Y. On the fourth day after seeding, medium in the upper compartment was replaced with differentiation media (Table S5), and medium in the lower compartment was replaced with MM (no Y). Media were changed daily for 4 days. On the eighth day after seeding, cells were either isolated for FACS (see below) or rinsed with PBS and fixed with 4% PFA (ThermoFisher Scientific AC416780250) for 15 min. Following fixation, cells were rinsed with PBS, permeabilized with 0.5% Triton X-100 (ThermoFisher Scientific AC215682500) in PBS for 20 min at room temperature, then rinsed twice with PBS +3% BSA (FisherScientific BP1600-1, diluted in PBS). Blocking was performed for 30 min at room temperature in 3% BSA. After blocking, cells were incubated with KRT20 primary antibody (1:200 dilution, Cell Signaling D9Z1Z) in 3% BSA for 48 h at 4°C, rinsed with 3% BSA, then incubated with a donkey anti-rabbit Cy3 secondary antibody (Jackson ImmunoResearch 711-166-152) and APC-conjugated KI67 antibody (Invitrogen 17-5698-83) for 1 h at room temperature. Cells were rinsed twice with 3% BSA, and nuclei were stained with bisbenzimide A (10 μg/mL, SigmaAldrich B1155) in 3% BSA for 5 min, then rinsed and stored in 3% BSA for microscopic analysis.

FACS, qPCR, and organoid formation

After growth and compartmentalization of PCM devices, culture media was aspirated and replaced with 4 mL warm PBS + Y, incubating for 5 min at 37°C. PBS was then aspirated, and 500 μL warm TrypLE was added to both the upper and lower compartments. Devices were incubated for 10 min at 37°C, pipetting vigorously after 5 and 10 min. Dislodged cells were then further dissociated with a 28G insulin syringe, and TrypLE was neutralized by adding 4 mL of culture media. Cells were pelleted at 800 × g for 3 min, resuspended in 1 mL PBS then re-pelleted. Cells were resuspended in MM + Y and sorted by FACS using an Aria II cell sorter (UNC Flow Cytometry Core). FSC-A, BSC-A, FSC-H, and BSC-H parameters were used to enrich for live single cells, and FSC-H and GFP parameters were used to sort populations expressing different levels of emGFP into cold MM + Y. For qPCR, RNA was isolated using the RNAqueous Micro Kit (Invitrogen AM1931). We performed cDNA amplification and qPCR with SsoAdvanced Universal Probe Supermix (Bio-Rad 1725281), using TaqMan probes Hs00197437_m1 (*OLFM4*), Hs00969442_m1 (*LGR5*), Hs04260396_g1 (*MKI67*), Hs00300643_m1 (KRT20), and Hs03003631_g1 (*18S*). To quantify organoid formation, 5,000 single cells (2,500 cells for negative population) were sorted into separate tubes and resuspended in 10 μL of ice-cold GFR Matrigel (Corning 354230) per 500 cells. We dispensed 10 μL aliquots of cells into separate wells of a 96-well culture plate, incubated for 25 min at 37°C to facilitate gel formation, then overlaid with 100 μL MM + Y. Media was changed every other day with fresh MM for 7 days, at which point organoids were fixed with pre-warmed 4% PFA for 15 min for quantification.

Statistics

Due to non-normality, statistical analysis of biological replicates in Figures 2B, 2C, 2D, 3F, and 5A were performed using a Mann-Whitney *U* test, and Figure 2E used a Kruskal-Wallis test. Analysis of technical replicates used *t*-tests, with Figures 4 and 7B using a Student *t*-test, Figure 7D using Welch's t test due to the expectation of unequal variance between samples, and qPCR data comparing Δ18S values between samples using a Student *t*-test.

SUPPLEMENTAL INFORMATION

Supplemental information can be found online at https://doi.org/10.1016/j.stemcr.2022.04.005.

AUTHOR CONTRIBUTIONS

Conceptualization, K.A.B and S.T.M.; Methodology, K.A.B. and S.T.M.; Validation, K.A.B., S.T.M., J.B., and M.T.O.; Formal Analysis, K.A.B.; Investigation, K.A.B., M.T.O., and N.P.K.; Resources, K.A.B., S.T.M., and I.G.; Data Curation, K.A.B.; Writing – Original Draft, K.A.B.; Writing – Review & Editing, S.T.M., J.B., and M.T.O.; Visualization, K.A.B. and M.T.O.; Supervision, S.T.M.; Project Administration, S.T.M. and K.A.B.; Funding Acquisition, S.T.M. and K.A.B.

CONFLICTS OF INTERESTS

S.T.M has a financial interest in Altis Biosystems Inc., which licenses the technology used in this study.

ACKNOWLEDGMENTS

We would like to thank the anonymous organ donors, their families, and HonorBridge (formerly Carolina Donor Services), Organ Procurement Organization, Durham, North Carolina, for providing the tissue samples used in this study. We would also like to thank UNC's Human Pluripotent Cell Core Facility for use of their Neon Transfection instrument, UNC's Advanced Analytics Core and Flow Cytometry Core for help with flow cytometry and FACS, and the UNC Animal Models Core for providing elements used in plasmid construction. Figure schematics were created with BioRender.com. This work was performed in part at the Chapel Hill Analytical and Nanofabrication Laboratory (CHANL) a member of the NC Research Triangle Nanotechnology Network (RTNN) which is supported by NSF Grant ECCS-2025064 as a part of the National Nanotechnology Coordinated Infrastructure (NNCI), the UNC Flow Cytometry Core Facility supported in part by P30 CA016086 Cancer Center Core Support Grant and by the North Carolina Biotech Center Institutional Support Grant 2012-IDG-1006, and the UNC Advanced Analytics Core supported by the Center for Gastrointestinal Biology and Disease (CGIBD) Core Grant P30DK034987. This work was funded by NIH grants T32GM133364 (K.A.B.), F30DK126307 (M.T.O.), F32DK124929 and T32DK07737 (J.B.), R01DK115806, R43DK125155, and R01DK109559 (S.T.M.), and the Katherine E. Bullard Charitable

Trust for Gastrointestinal Stem Cell and Regenerative Research (S.T.M.). The UNC IRB determined this study does not constitute human subjects research.

Received: October 6, 2021
Revised: April 7, 2022
Accepted: April 8, 2022
Published: May 5, 2022

REFERENCES

Barker, N. (2014). Adult intestinal stem cells: critical drivers of epithelial homeostasis and regeneration. Nat. Rev. Mol. Cell Biol. *15*, 19–33. https://doi.org/10.1038/nrm3721.

Barker, N., van Es, J.H., Kuipers, J., Kujala, P., van den Born, M., Cozijnsen, M., Haegebarth, A., Korving, J., Begthel, H., Peters, P.J., and Clevers, H. (2007). Identification of stem cells in small intestine and colon by marker gene Lgr5. Nature *449*, 1003–1007. https://doi.org/10.1038/nature06196.

Brinkman, E.K., Chen, T., Amendola, M., and van Steensel, B. (2014). Easy quantitative assessment of genome editing by sequence trace decomposition. Nucleic Acids Res. *42*, e168. https://doi.org/10.1093/nar/gku936.

Burclaff, J., Bliton, R.J., Breau, K.A., Ok, M.T., Gomez-Martinez, I., Ranek, J.S., Bhatt, A.P., Purvis, J.E., Woosley, J.T., and Magness, S.T. (2022). A proximal-to-distal survey of healthy adult human small intestine and colon epithelium by single-cell transcriptomics. Cell. Mol. Gastroenterol. Hepatol. *13*, 1554–1589.

Busslinger, G.A., Weusten, B.L.A., Bogte, A., Begthel, H., Brosens, L.A.A., and Clevers, H. (2021). Human gastrointestinal epithelia of the esophagus, stomach, and duodenum resolved at single-cell resolution. Cell Rep. *34*, 108819. https://doi.org/10.1016/j.celrep.2021.108819.

de Carvalho, T.G., Pellenz, F.M., Laureano, A., da Rocha Silla, L.M., Giugliani, R., Baldo, G., and Matte, U. (2018). A simple protocol for transfecting human mesenchymal stem cells. Biotechnol. Lett. *40*, 617–622. https://doi.org/10.1007/s10529-018-2505-8.

Cheng, H., and Leblond, C.P. (1974). Origin, differentiation and renewal of the four main epithelial cell types in the mouse small intestine I. Columnar cell. Am. J. Anat. *141*, 461–479. https://doi.org/10.1002/aja.1001410403.

Cong, L., Ran, F.A., Cox, D., Lin, S., Barretto, R., Habib, N., Hsu, P.D., Wu, X., Jiang, W., Marraffini, L.A., and Zhang, F. (2013). Multiplex genome engineering using CRISPR/Cas systems. Science *339*, 819–823. https://doi.org/10.1126/science.1231143.

van der Flier, L.G., Haegebarth, A., Stange, D.E., van de Wetering, M., and Clevers, H. (2009). OLFM4 is a robust marker for stem cells in human intestine and marks a subset of colorectal cancer cells. Gastroenterology *137*, 15–17. https://doi.org/10.1053/j.gastro.2009.05.035.

Fujii, M., Matano, M., Nanki, K., and Sato, T. (2015). Efficient genetic engineering of human intestinal organoids using electroporation. Nat. Protoc. *10*, 1474–1485. https://doi.org/10.1038/nprot.2015.088.

Gersemann, M., Becker, S., Nuding, S., Antoni, L., Ott, G., Fritz, P., Oue, N., Yasui, W., Wehkamp, J., and Stange, E.F. (2012). Olfactomedin-4 is a glycoprotein secreted into mucus in active IBD. J. Crohns Colitis *6*, 425–434. https://doi.org/10.1016/j.crohns.2011.09.013.

Hinman, S.S., Wang, Y., Kim, R., and Allbritton, N.L. (2021). In vitro generation of self-renewing human intestinal epithelia over planar and shaped collagen hydrogels. Nat. Protoc. *16*, 352–382. https://doi.org/10.1038/s41596-020-00419-8.

Honn, K.V., Singley, J.A., and Chavin, W. (1975). Fetal bovine serum: a multivariate standard. Proc. Soc. Exp. Biol. Med. *149*, 344–347. https://doi.org/10.3181/00379727-149-38804.

Jordan, E.T., Collins, M., Terefe, J., Ugozzoli, L., and Rubio, T. (2008). Optimizing electroporation conditions in primary and other difficult-to-transfect cells. J. Biomol. Technol. JBT *19*, 328–334.

Kashfi, H., Jinks, N., and Nateri, A.S. (2020). Generating and utilizing murine Cas9-expressing intestinal organoids for large-scale knockout genetic screening. Methods Mol. Biol. *2171*, 257–269. https://doi.org/10.1007/978-1-0716-0747-3_17.

Kiela, P.R., and Ghishan, F.K. (2016). Physiology of intestinal absorption and secretion. Best Pract. Res. Clin. Gastroenterol. *30*, 145–159. https://doi.org/10.1016/j.bpg.2016.02.007.

Kim, R., Wang, Y., Hwang, S.-H.J., Attayek, P.J., Smiddy, N.M., Reed, M.I., Sims, C.E., and Allbritton, N.L. (2018). Formation of arrays of planar, murine, intestinal crypts possessing a stem/proliferative cell compartment and differentiated cell zone. Lab. Chip *18*, 2202–2213. https://doi.org/10.1039/c8lc00332g.

Kucia, M., Reca, R., Campbell, F.R., Zuba-Surma, E., Majka, M., Ratajczak, J., and Ratajczak, M.Z. (2006). A population of very small embryonic-like (VSEL) CXCR4(+)SSEA-1(+)Oct-4+ stem cells identified in adult bone marrow. Leukemia *20*, 857–869. https://doi.org/10.1038/sj.leu.2404171.

Liang, X., Potter, J., Kumar, S., Zou, Y., Quintanilla, R., Sridharan, M., Carte, J., Chen, W., Roark, N., Ranganathan, S., et al. (2015). Rapid and highly efficient mammalian cell engineering via Cas9 protein transfection. J. Biotechnol. *208*, 44–53. https://doi.org/10.1016/j.jbiotec.2015.04.024.

Mali, P., Yang, L., Esvelt, K.M., Aach, J., Guell, M., DiCarlo, J.E., Norville, J.E., and Church, G.M. (2013). RNA-guided human genome engineering via Cas9. Science *339*, 823–826. https://doi.org/10.1126/science.1232033.

Matano, M., Date, S., Shimokawa, M., Takano, A., Fujii, M., Ohta, Y., Watanabe, T., Kanai, T., and Sato, T. (2015). Modeling colorectal cancer using CRISPR-Cas9–mediated engineering of human intestinal organoids. Nat. Med. *21*, 256–262. https://doi.org/10.1038/nm.3802.

McCoy, A.M., Collins, M.L., and Ugozzoli, L.A. (2010). Using the gene pulser MXcell electroporation system to transfect primary cells with high efficiency. J. Vis. Exp. *35*, 1662. https://doi.org/10.3791/1662.

Merlos-Suárez, A., Barriga, F.M., Jung, P., Iglesias, M., Céspedes, M.V., Rossell, D., Sevillano, M., Hernando-Momblona, X., da Silva-Diz, V., Muñoz, P., et al. (2011). The intestinal stem cell signature identifies colorectal cancer stem cells and predicts disease relapse. Cell Stem Cell *8*, 511–524. https://doi.org/10.1016/j.stem.2011.02.020.

Moll, R., Schiller, D.L., and Franke, W.W. (1990). Identification of protein IT of the intestinal cytoskeleton as a novel type I cytokeratin with unusual properties and expression patterns. J. Cell Biol. *111*, 567–580. https://doi.org/10.1083/jcb.111.2.567.

Pai, J.-H., Wang, Y., Salazar, G.T., Sims, C.E., Bachman, M., Li, G.P., and Allbritton, N.L. (2007). Photoresist with low fluorescence for bioanalytical applications. Anal. Chem. *79*, 8774–8780. https://doi.org/10.1021/ac071528q.

De Paiva, C.S., Pflugfelder, S.C., and Li, D.-Q. (2006). Cell size correlates with phenotype and proliferative capacity in human corneal epithelial cells. Stem Cells *24*, 368–375. https://doi.org/10.1634/stemcells.2005-0148.

Prasanna, G.L., and Panda, T. (1997). Electroporation: basic principles, practical considerations and applications in molecular biology. Bioproc. Eng. *16*, 261–264. https://doi.org/10.1007/s004490050319.

Sato, T., Vries, R.G., Snippert, H.J., van de Wetering, M., Barker, N., Stange, D.E., van Es, J.H., Abo, A., Kujala, P., Peters, P.J., and Clevers, H. (2009). Single Lgr5 stem cells build crypt-villus structures in vitro without a mesenchymal niche. Nature *459*, 262–265. https://doi.org/10.1038/nature07935.

Sato, T., Stange, D.E., Ferrante, M., Vries, R.G.J., van Es, J.H., van den Brink, S., van Houdt, W.J., Pronk, A., van Gorp, J., Siersema, P.D., and Clevers, H. (2011). Long-term expansion of epithelial organoids from human colon, adenoma, adenocarcinoma, and Barrett's epithelium. Gastroenterology *141*, 1762–1772. https://doi.org/10.1053/j.gastro.2011.07.050.

Schwank, G., and Clevers, H. (2016). CRISPR/Cas9-Mediated genome editing of mouse small intestinal organoids. Methods Mol. Biol. *1422*, 3–11. https://doi.org/10.1007/978-1-4939-3603-8_1.

Stemmer, M., Thumberger, T., del Sol Keyer, M., Wittbrodt, J., and Mateo, J.L. (2015). CCTop: an intuitive, flexible and reliable CRISPR/Cas9 target prediction tool. PLoS One *10*, e0124633. https://doi.org/10.1371/journal.pone.0124633.

Wang, Y., DiSalvo, M., Gunasekara, D.B., Dutton, J., Proctor, A., Lebhar, M.S., Williamson, I.A., Speer, J., Howard, R.L., Smiddy, N.M., et al. (2017a). Self-renewing monolayer of primary colonic or rectal epithelial cells. Cell. Mol. Gastroenterol. Hepatol. *4*, 165–182.e7. https://doi.org/10.1016/j.jcmgh.2017.02.011.

Wang, Y., Gunasekara, D.B., Reed, M.I., DiSalvo, M., Bultman, S.J., Sims, C.E., Magness, S.T., and Allbritton, N.L. (2017b). A microengineered collagen scaffold for generating a polarized crypt-villus architecture of human small intestinal epithelium. Biomaterials *128*, 44–55. https://doi.org/10.1016/j.biomaterials.2017.03.005.

Wang, Y., Kim, R., Sims, C.E., and Allbritton, N.L. (2019). Building a thick mucus hydrogel layer to improve the physiological relevance of in vitro primary colonic epithelial models. Cell. Mol. Gastroenterol. Hepatol. *8*, 653–655.e5. https://doi.org/10.1016/j.jcmgh.2019.07.009.

Stem Cell Reports, Volume *17*

Supplemental Information

Efficient transgenesis and homology-directed gene targeting in monolayers of primary human small intestinal and colonic epithelial stem cells

Keith A. Breau, Meryem T. Ok, Ismael Gomez-Martinez, Joseph Burclaff, Nathan P. Kohn, and Scott T. Magness

Supplemental Table 1. Electrical pulse parameters for optimization

Sample #	Parameters			Transfection Efficiency			Cell Viability		
	Voltage	Length (ms)	# Pulses	Duo	Jej	Ile	Duo	Jej	Ile
1	0	0	0	Neg	Neg	Neg	High	High	High
2	1400	20	1	SubLow	Low	Moderate	High	High	High
3	1500	20	1	Low	Low	Moderate	Low	High	High
4	1600	20	1	Moderate	Moderate	High	High	High	High
5	1700	20	1	Moderate	High	High	High	High	High
6	1100	30	1	Neg	Neg	Neg	High	High	High
7	1200	30	1	SubLow	SubLow	Low	High	High	Moderate
8	1300	30	1	Low	SubLow	Moderate	High	High	Low
9	1400	30	1	Moderate	Moderate	Moderate	Low	High	Low
10	1000	40	1	Neg	Neg	SubLow	High	High	Moderate
11	1100	40	1	SubLow	Neg	SubLow	Moderate	High	Low
12	1200	40	1	Low	SubLow	Low	Moderate	High	SubLow
13	1100	20	2	SubLow	SubLow	SubLow	High	High	Low
14	1200	20	2	SubLow	SubLow	SubLow	SubLow	High	Low
15	1300	20	2	Moderate	Low	Moderate	SubLow	High	SubLow
16	1400	20	2	High	Moderate	Moderate	SubLow	Moderate	Neg
17	850	30	2	Neg	Neg	Neg	Low	High	SubLow
18	950	30	2	Neg	Neg	Neg	Low	High	SubLow
19	1050	30	2	SubLow	SubLow	Neg	Low	High	Neg
20	1150	30	2	Low	Moderate	SubLow	Low	Moderate	Neg
21	1300	10	3	Neg	Neg	Neg	Moderate	High	SubLow
22	1400	10	3	SubLow	SubLow	Neg	Low	High	SubLow
23	1500	10	3	SubLow	Low	SubLow	Low	High	Low
24	1600	10	3	High	Moderate	Low	SubLow	Moderate	Neg

Supplemental Table 2. Organ donor demographics

Identifier	Patient Sex	Patient Age	Race/Ethnicity	Cause of Death
Donor 1	M	12 years	White	Cerebrovascular/Stroke
Donor 2	M	34 years	Hispanic	Anoxia, Asphyxiation (unknown downtime)
Donor 3	F	62 years	White	Cardiovascular, Death from Natural Causes

Supplemental Table 3. CCTop off-target loci for *OLFM4* gRNA

gRNA Sequence:	ACTTACTTAGATATCTGCAG							
Coordinates	strand	MM	target_seq	PAM	distance		gene name	gene id
chr13:53050828-53050850	+	0	ACTTACTT [AGATATCTGCAG]	GGG	0	E	OLFM4	ENSG00000102837
chr4:95984460-95984482	-	4	TAACACTT [AGATATCTGCAG]	AAG	NA	-	NA	NA
chr6:119536897-119536919	-	4	AGTAGGTT [AGATATCTGCAG]	TGG	NA	-	NA	NA
chr10:1497775-1497797	+	4	CATTCCTG [AGATATCTGCAG]	AAG	28840	I	ADARB2-AS1	ENSG00000205696
chr4:158333757-158333779	+	4	ATCTATGT [AGATATCTGCAG]	GGG	18287	I	RXFP1	ENSG00000171509
chr12:105189620-105189642	-	4	GTTTAGTA [AGATATCTGCAG]	TGG	130	I	APPL2	ENSG00000136044
chr2:127745290-127745312	+	4	GGTTAATC [AGATATCTGCAG]	CAG	7236	I	WDR33	ENSG00000136709
chr5:61465479-61465501	-	3	ACTAATTT [GGATATCTGCAG]	TGG	7180	I	ZSWIM6	ENSG00000130449
chr2:219136091-219136113	-	4	ACAAAATC [AGATATCTGCAG]	GGG	10431	I	NHEJ1	ENSG00000187736
chr3:115685944-115685966	+	4	ATTTTATC [AGATATCTGCAG]	CGG	9334	I	GAP43	ENSG00000172020
chr21:21940614-21940636	-	4	TTTTACCT [TGATATCTGCAG]	TAG	7015	I	AP000472.3	ENSG00000227075
chr10:114724009-114724031	+	4	ATTCATTT [TGATATCTGCAG]	GAG	33375	-	PPIAP19	ENSG00000228169
chr18:47316063-47316085	+	3	ACTTCATT [AAATATCTGCAG]	TGG	30266	I	CTD-2130O13.1	ENSG00000267761
chr6:81765343-81765365	-	4	ATTTGCCC [AGATATCTGCAG]	CAG	840	-	RP5-991C6.2	ENSG00000219702
chr3:128045637-128045659	+	4	ACAGATTT [TGATATCTGCAG]	AGG	5982	-	SEC61A1	ENSG00000058262
chr14:42710344-42710366	-	4	ACAGATTT [CGATATCTGCAG]	GGG	6689	-	CTD-2307P3.1	ENSG00000258394
chr4:144546999-144547021	-	4	ACAGATTT [TGATATCTGCAG]	AGG	25217	-	KRT18P51	ENSG00000250504
chr6:101110566-101110588	+	4	ATTTAGAA [AGATATCTGCAG]	GAG	NA	-	NA	NA
chr13:39413026-39413048	-	4	ATCTACTG [TGATATCTGCAG]	AAG	34500	I	LHFP	ENSG00000183722
chr2:56417840-56417862	-	4	AGAGACTT [AGTTATCTGCAG]	TAG	31667	-	CCDC85A	ENSG00000055813

Supplemental Table 4. Primers used in this study

Primer Name	Oligo Sequence (5' to 3')	Purpose
KB085_Olfm4-ScF2	CCGTACTATGAACACCAGAACAG	PCR amplification of *OLFM4* gRNA target region for sequencing
KB086_Olfm4-ScR2	CGCCATACTGAGTTTTAAGTTCC	PCR amplification of *OLFM4* gRNA target region for sequencing
KB026_Olfm4-SqF1	AGCATTAACTATAACCCTTTTGAC	Sequencing primer for *OLFM4* gRNA target region
KB069_Olfm4-5HR-F2	tggccgattcattaatgcagGACTGAATGCACTGAACTAAGAGG	Amplification of OLFM4 5' homology region
KB038_Olfm4-5HR-R	tcgaatagcttggctgcaggAGATATCTAAGTAAGTGGAGAAGAC	Amplification of OLFM4 5' homology region
KB039_Olfm4-3HR-F	ttaggtccctcgaggggatcGCAGGGGTGTCTAAAAGTGTG	Amplification of OLFM4 3' homology region
KB040_Olfm4-3HR-R	cctcttcgctattacgccagAAGGTTTCCAACTACTGCACTG	Amplification of OLFM4 3' homology region
KB045_emGFP-F	TCGACCTGCAGCCAAGCTATTCGAATTCCCCCCCCCCCTAACGTTACTG	Amplification of emGFP coding sequence
KB046_emGFP-R	TGATCGGAATTGGGCTGCAGGAATTCTTACTTGTACAGCTCGTCCATGC	Amplification of emGFP coding sequence
KB081_IRES-SqR1	TAACATATAGACAAACGCACACC	Screening for OLFM4-emGFP desired integration event
KB084_bGHpA-SqF1	ATGCGGTGGGCTCTATGG	Screening for OLFM4-emGFP desired integration event
KB097_Olfm4-5LR-F1	CTGTAGAGCTGACATTGGGTTTGC	Screening for OLFM4-emGFP desired integration event
KB098_Olfm4-3LR-R1	AGGTAGCCATTCTCCCAACCTC	Screening for OLFM4-emGFP desired integration event

Supplemental Table 5. Medias and buffers used in this study

L-WRN Collection Medium

Reagent	Concentration	Vendor / Cat#
Adv. DMEM/F12	78% v/v	Gibco 12634010
Fetal Bovine Serum	20% v/v	VWR Premium 97068-085
GlutaMax	2 mM (1X)	Gibco 35050061
Pen/Strep	1X	Gibco 15070063

2x hISC Basal Medium

Reagent	Concentration	Vendor / Cat#
Adv. DMEM/F12	89% v/v	Gibco 12634010
B-27 Supplement	4% v/v	Gibco 12587001
Nicotinamide	10 mM	Sigma-Aldrich N0636
HEPES	20 mM	Corning 25-060-Cl
Glutamax	2 mM (1X)	Gibco 35050061
Pen/Strep	1X	Gibco 15070063
N-Acetylcysteine	1.25 mM	Sigma-Aldrich A9165
Primocin	50 ug/mL	Invivogen ant-pm-05
SB202190	3 uM	Peprotech 1523072
mEGF	50 ng/mL	Peprotech 315-09
Gastrin	2 nM	Sigma-Aldrich G9145
Prostaglandin E2	10 nM	Peprotech 3632464

Isolation Buffer

Reagent	Concentration	Vendor / Cat#
Na_2HPO_4	5.6 mM	Sigma S7907
KH_2PO_4	8.0 mM	Sigma P5655
NaCl	96.2 mM	Sigma S5886
KCl	1.6 mM	Sigma P5405
Sucrose	43.4 mM	Fisher BP 220-1
d-sorbitol	54.9 mM	Fisher BP439-500
Y27632	100 uM	Selleck Chemical S6390

Differentiation Medium

Reagent	Concentration	Vendor / Cat#
Adv. DMEM/F12	89% v/v	Gibco 12634010
HEPES	10 mM	Corning 25-060-Cl
Glutamax	2 mM (1X)	Gibco 35050061
Pen/Strep	1X	Gibco 15070063
N-Acetylcysteine	1.25 mM	Sigma-Aldrich A9165
Primocin	50 ug/mL	Invivogen ant-pm-05
mEGF	50 ng/mL	Peprotech 315-09
A83-01	500 uM	Sigma-Aldrich SML0788

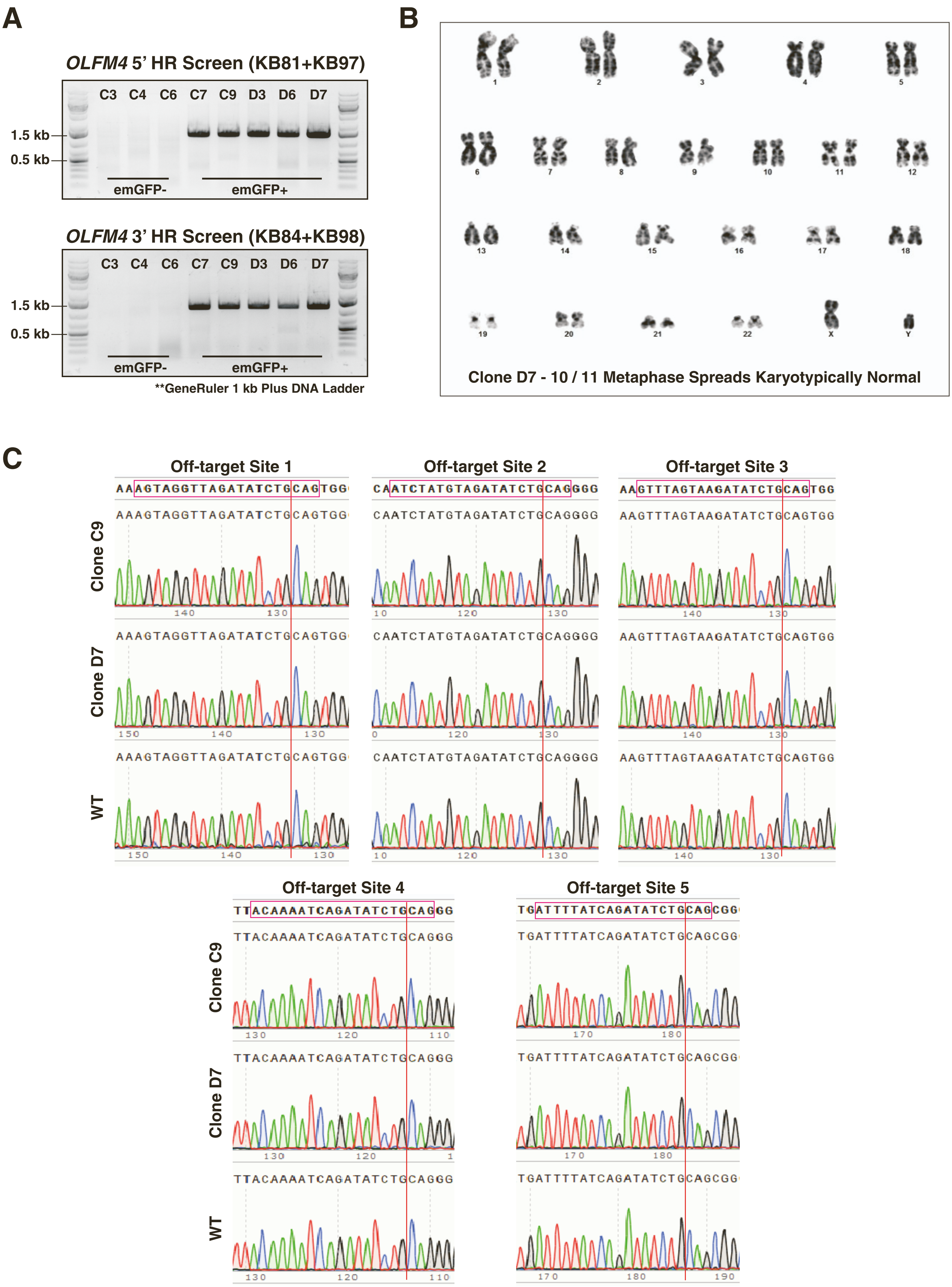

Supplemental Figure 1

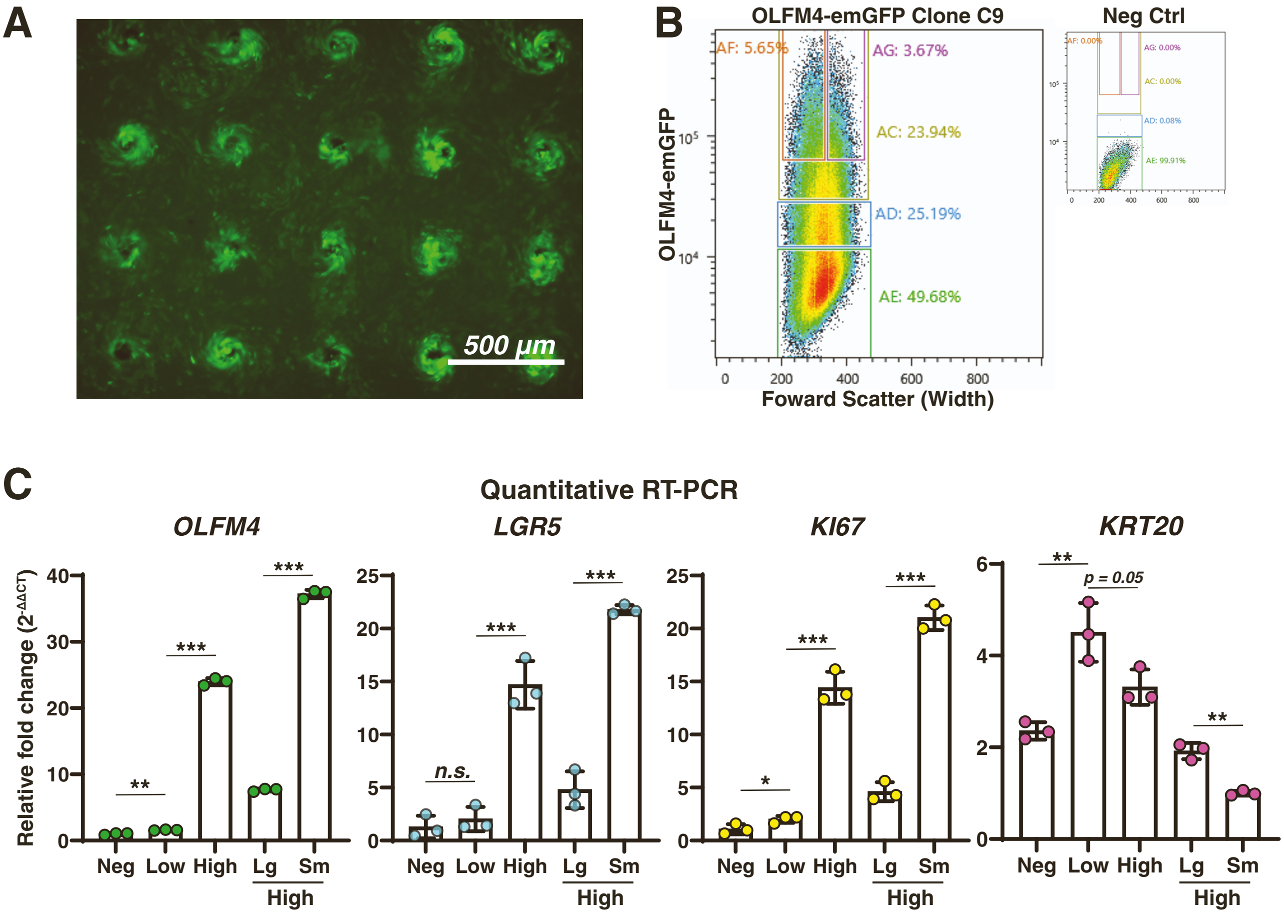

Supplemental Figure 2

Supplemental Table 1, related to Figure 1. Electrical parameters used to optimize electroporation efficiency. Twenty-four parameter sets were tested, varying electrical voltage, pulse duration, and pulse number. Transfection efficiency and cell viability of three biological replicates were estimated and binned into 5 categories (Neg, SubLow, Low, Moderate, and High). Condition #5 (1700 V, 20 ms, 1 pulse), highlighted, was used for further experiments.

Supplemental Table 2, related to Figure 2. Demographic details of organ donors used to test biological reproducibility of transfection efficiency.

Supplemental Table 3, related to Figure 5. CCTop output of the selected *OLFM4*-targetting gRNA, showing DNA sequences throughout the genome with homology to the gRNA sequence. First entry is the desired cleavage site in the terminal exon of *OLFM4*. Latter entries are potential off-target cleavage sites, ranked by decreasing predicted risk of cleavage, with mismatched nucleotides shown in red. gRNA was chosen such that all potential off-target sites have at least four mismatches, or three mismatches with at least one mismatch within the 12 bp “seed” region (shown in brackets), proximal to the Cas9 cleavage site.

Supplemental Table 4, related to Figure 5. Primers used for generating TIDE results (top), generating the *OLFM4* targeting plasmid (middle), and validating integration of the IRES-emGFP sequence (bottom).

Supplemental Table 5. Isolation buffer used for crypt isolation, and medias used for subsequent hISC expansion and differentiation.

Supplemental Figure 1, related to Figure 5. (A) PCR validation of IRES-emGFP integration at the *OLFM4* locus. Three emGFP-negative clones were included for a negative control, along with 5 emGFP+ clones. Primers were designed to span the homology arms used for HDR, with one primer in each screen binding to the native *OLFM4* locus outside of the homology regions and the other binding to the IRES-emGFP insert. **(B)** Example metaphase spread for OLFM4-emGFP

clone D7, which is used in the primary figures of the manuscript, demonstrating these cells are karyotypically normal. **(C)** Sequencing results of high-risk off-target cleavage sites of the OLFM4 gRNA. Sites represent all genomic loci with a perfect match of the gRNA seed region (12 bp at 3' end) and a PAM sequence of NGG. Sequencing demonstrates no mutations in either clone used in this study.

Supplemental Figure 2, related to Figure 6. Analysis of a second OLFM4-emGFP clone (clone C9) demonstrates similar results to clone D7, showing **(A)** compartmentalization of emGFP+ and emGFP- zones on a PCM device, **(B)** flow cytometry of PCM-isolated cells, with a gradient of OLFM4-emGFP signal and a separate, smaller OLFM4-emGFP^{high} population, and **(C)** qPCR results of FACS-isolated cell populations demonstrating High^{sm} cells highly express stem cell markers *OLFM4*, *LGR5*, and *KI67*, and poorly express differentiation marker *KRT20*.
